# Similarity Selection and the Evolution of Sex: Revisiting the Red Queen

**DOI:** 10.1371/journal.pbio.0040265

**Published:** 2006-08-01

**Authors:** Aneil F Agrawal

**Affiliations:** **1**Department of Ecology and Evolutionary Biology, University of Toronto, Toronto, Ontario, Canada; University of EdinburghUnited Kingdom

## Abstract

For over 25 years, many evolutionary ecologists have believed that sexual reproduction occurs because it allows hosts to change genotypes each generation and thereby evade their coevolving parasites. However, recent influential theoretical analyses suggest that, though parasites can select for sex under some conditions, they often select against it. These models assume that encounters between hosts and parasites are completely random. Because of this assumption, the fitness of a host depends only on its own genotype (“genotypic selection”). If a host is even slightly more likely to encounter a parasite transmitted by its mother than expected by random chance, then the fitness of a host also depends on its genetic similarity to its mother (“similarity selection”). A population genetic model is presented here that includes both genotypic and similarity selection, allowing them to be directly compared in the same framework. It is shown that similarity selection is a much more potent force with respect to the evolution of sex than is genotypic selection. Consequently, similarity selection can drive the evolution of sex even if it is much weaker than genotypic selection with respect to fitness. Examination of explicit coevolutionary models reveals that even a small degree of mother–offspring parasite transmission can cause parasites to favor sex rather than oppose it. In contrast to previous predictions, the model shows that weakly virulent parasites are more likely to favor sex than are highly virulent ones. Parasites have figured prominently in discussions of the evolution of sex, but recent models suggest that parasites often select against sex rather than for it. With the inclusion of small and realistic exposure biases, parasites are much more likely to favor sex. Though parasites alone may not provide a complete explanation for sex, the results presented here expand the potential for parasites to contribute to the maintenance of sex rather than act against it.

## Abstract 2

That parasites help explain why sex evolved has been disputed, but a new model incorporating similarity selection--where a host encounters parasites from its mother more often than by chance--makes its evolution more likely.

## Introduction

The Red Queen hypothesis posits that host–parasite co-evolution plays an important role in the evolution of genetic mixing, e.g., segregation, recombination, and sex. Indeed, a number of empirical studies report patterns consistent with this idea [ 1– 6]. Although the Red Queen hypothesis has been popular for over two decades [ 7– 10], only recently have coevolutionary models focused on gene-level advantages of genetic mixing rather than group-level advantages [ 11– 13]. Surprisingly, these models suggest that, under many conditions, parasites select against genes that increase the degree of genetic mixing.

These host–parasite models are closely related to previously developed single-species models that do not specify the ecological source of selection. For example, Barton [ 14] used a single-species model to identify the conditions favoring recombination in a large panmictic population. These conditions are restrictive: epistasis must be both negative and weak. Using a haploid host–parasite model, Otto and Nuismer [ 12] showed that Barton's result holds when parasites are the source of selection. Moreover, they found that parasites typically generate epistasis that is too strong and, consequently, select against sex and recombination. In diploids, sex may be favored because of segregation rather than recombination. Using a single-species diploid model, Otto [ 15] showed that an advantage to sex through segregation exists only when selected alleles display a specific level of dominance. Another study [ 11] found the same result when parasites were the source of selection and showed that parasites typically produce dominance values that are too strong to favor sex in panmictic populations.

The concordance between single-species models and host–parasite models is not unexpected. From the perspective of a genetic modifier of genetic mixing, the ecological force (parasites or otherwise) is irrelevant—all that matters is the pattern of selection experienced by loci in the genome. Consequently, we can use a general single-species model to determine how a modifier evolves under various types of selection and then evaluate different ecological forces by calculating the type of selection they produce in terms of the selection parameters used in the single-species model. This approach requires that the framework used to describe fitness in the single-species model is sufficiently general that it can properly depict the pattern of selection generated by the ecological force of interest.

To date, single-species modifier models have assumed that an individual's fitness depends only on its own genotype. I refer to this type of fitness framework as “genotypic selection only” because it involves only selection acting directly on an individual's own genotype. Genotypic selection is sufficient for describing many types of selection, including selection by parasites, provided that hosts encounter parasites at random. In any generation, selection coefficients affecting host fitness can be calculated as functions of virulence and the current distribution of parasite genotype frequencies. However, if hosts do not encounter parasites completely at random, then a model with only genotypic selection is inadequate.

In reality, hosts are likely to be biased towards encountering parasites produced by their mothers. The degree of bias will depend on the ecology of host and pathogen. At one extreme, the bias will be strong if there is direct vertical transmission in the traditional sense, e.g., parasites move directly from an infected mother to her eggs or altricial offspring. For example, many, perhaps most, phytopathogenic bacteria are capable of seed transmission [ 16]. In contrast, the bias will be weak if offspring disperse widely and transmission occurs after offspring are independent of their mothers. Even in such cases, some bias is expected simply because an offspring has a greater than random chance of being in some place near where its infected mother has been. A host will be more likely to encounter a parasite transmitted by its mother than one transmitted by some other infected host from the opposite end of the species range. This logic implies that exposure bias may occur even in systems with parasites having indirect life cycles, provided that the other host species involved in the parasite's life cycle do not move very large distances from the site of infection. Though exposure bias may be very small in systems with indirect parasites and/or wide offspring dispersal, we cannot determine what level of bias can be regarded as effectively negligible without formal analysis.

Given that hosts are genetically variable in their susceptibility to different parasite genotypes, an offspring that has exactly the same genotype as its mother will tend to be more susceptible to parasites produced by its infected mother than will a sibling with a somewhat different genotype. To the extent that hosts encounter parasites produced by their mothers, the fitness of an individual will depend not only on its own genotype (genotypic selection) but also on the degree of similarity to its mother's genotype, i.e., similarity selection.

The idea that parasites may generate selection against offspring having exactly the same genome as their mothers is present in some verbal descriptions of the Red Queen and is discussed at length by Rice [ 17] (see also [ 18]). It is intuitive that similarity selection will generate selection for genetic mixing. However, there has been no attempt to quantify how powerful such selection is relative to genotypic selection in shaping the evolution of genetic mixing. Here I present analytical results from a single-species diploid model that includes both genotypic and similarity selection (described in detail in Protocol S1). I then analyze some simple host–parasite models to determine the amount of genotypic and similarity selection produced by coevolutionary models (described in detail in Protocol S2). Computer simulations are used to show how transmission bias alters the direction of selection on sex caused by parasites (described in detail in Protocol S3). Finally, I present analytical results from a single-species haploid model to demonstrate that although recombination and sex are favored under the same conditions with genotypic selection, this is not the case with similarity selection.

## Model and Results

### Diploid Two-Locus Model

This model involves two di-allelic loci in a diploid organism and is described in detail in Protocol S1. The **A** locus affects fitness; the modifier locus, **M**, affects the amount of sex. With respect to the fitness locus **A**, there are seven different types of offspring–mother combinations, e.g., *AA* offspring from *AA* mothers, *AA* offspring from *Aa* mothers, etc. (For simplicity, it is assumed there is no mutation so that an *AA* mother cannot produce an *aa* offspring and an *aa* mother cannot produce an *AA* offspring.) Six selection parameters are required to create a general fitness function capable of describing any pattern of fitness relationships among these seven offspring–mother combinations. Though the fitness function could be modeled in a number of ways, the fitness function used here is parameterized to represent three types of selection: genotypic selection, similarity selection, and maternal selection.

Genotypic selection refers to the effects of an individual's own genotype on its fitness. Here it is measured by *α _a_* and *ι* **_A_**. The parameter *α _a_* measures the reduction in fitness caused by carrying a single copy of the *a* allele; *ι* **_A_** is a multiplicative measure of dominance at the **A** locus. Let *w _G_* be the fitness of an individual when considering only genotypic selection (as was done in [ 15]). The values of *w _G_* for genotypes *AA*, *Aa*, and *aa* are 1, 1 − *α _a_*, and (1 − *α _a_*) ^2^ + *ι* **_A_**, respectively.

Similarity selection refers to the fitness effects of an individual having the same genotype as its mother. It is measured by the parameters *γ* **_A_** *, κ* **_A_** *_,a_,* and *κ* **_A_** *_,aa_*. The parameter *γ* **_A_** measures the reduction in fitness to an individual if it has the same genotype at the **A** locus as its mother, regardless of what this genotype is. The parameter *κ* **_A_** *_,a_* measures the additional reduction in fitness to an individual that carries at least one copy of the *a* allele and shares the same genotype at the **A** locus as its mother. The parameter *κ* **_A_** *_,aa_* measures the additional reduction in fitness to an individual that is homozygous for the *a* allele and shares the same genotype at the **A** locus as its mother (i.e., its mother is also *aa*). Let *w _S_* be the fitness of an individual when considering only similarity selection. An offspring of any genotype will have *w _S_* = 1 if its mother's genotype at the **A** locus is not the same as its own. When produced by mothers with the same genotype as their own, the values of *w _S_* for *AA*, *Aa*, and *aa* individuals are 1 − *γ* **_A_**, (1 − *γ* **_A_) **(1 − *κ* **_A_** *_,a_*), and (1 − *γ* **_A_) **(1 − *κ* **_A_** *_,a_*) ^2^(1 − *κ* **_A_** *_,aa_*), respectively.

Finally, the effect of the mother's genotype alone on the fitness of her offspring (“maternal selection”) is measured by *β _a_*. Let *w _M_* be the fitness of an individual when considering only maternal selection. An individual's value for *w _M_* depends only on its mother's genotype. Offspring from *AA, Aa,* and *aa* mothers have values of *w _M_* of 1, 1 − *β _a_*, and (1 − *β _a_*) ^2^, respectively. Though maternal selection has been included for the sake of completeness, this component of selection is unimportant to the results reported, and some readers may choose to ignore it.

Combining all three forms of selection, the fitness of an individual is given by



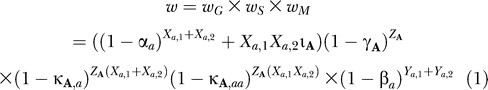



where *X _a,i_, Y _a,i_,* and *Z* **_A_** are indicator variables whose values are determined, respectively, by the individual's own genotype, its mother's genotype, and the similarity between the two (see Table 1 for a summary of parameter and variable definitions).

**Table 1:**
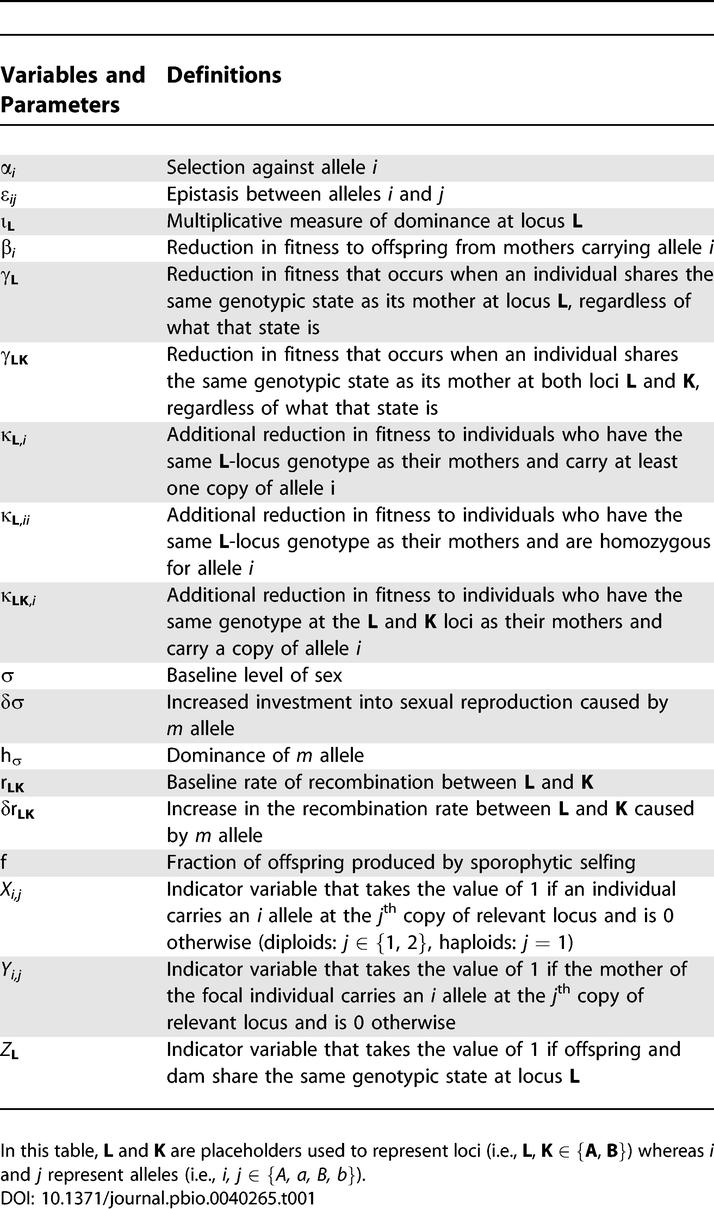
Model Parameters and Variables

In the discussion that follows, the term “ *strength* of selection” refers to the amount that genotypic or similarity selection affects the fitness of individuals; selection strength is determined by the magnitude of the selection parameters in Equation 1. The term “ *potency* of selection” refers to capacity for genotypic or similarity selection of a given strength to affect the evolution of sex, i.e., evolution at the **M** locus.

Reproduction occurs following selection. Individuals produce a fraction of their offspring sexually and the remainder asexually. The fraction produced sexually for *MM, Mm,* and *mm* individuals is *σ, σ + h _σ_δσ *, and *σ + δσ*, respectively. For the sake of discussion, we will assume that the *m* allele increases the amount of sex, i.e., *h _σ_*, *δσ* > 0. Of those offspring produced sexually, a fraction *f* are produced through sporophytic selfing. Sporophytic selfing is modeled here rather than gametophytic selfing as in [ 15] because sporophytic selfing should provide a better approximation to inbreeding that arises from population structure. The selfing rate does not depend on genotype.

The model follows the approach of other modifier models [ 14, 15] in employing the quasi-linkage equilibrium (QLE; [ 19]). This technique assumes that genetic associations (disequilibria) are close to their steady state values, which requires that selection be weak relative to the baseline level of genetic mixing. This type of analysis can be used to model coevolution when virulence is not too high [ 12], but does not apply to situations with very rapid fluctuations in non-linear genotypic selection (i.e., dominance or epistasis) that can occur with high levels of virulence under particular genetic architectures (simulations are used to examine such scenarios). One key difference between this and previous models is in the type of genetic associations that are necessary to fully describe the population. In models that involve only genotypic selection, the only genetic associations needed are associations among alleles within offspring. With the addition of similarity selection, it is also necessary to measure associations among alleles within mothers and, most importantly, associations between alleles that occur in offspring and those that occur in their mothers. In this model, there are seven associations involving genes within offspring, seven associations involving genes within their mothers, and 81 associations involving genes across the two generations.

It is assumed that genotypic selection is weak, but that similarity and maternal selection are much weaker. (Maternal and similarity selection are likely to be weaker than genotypic selection unless hosts are strongly biased towards encountering parasites transmitted by their mothers.) It is also assumed that the effect of the modifier is small and that the rate of selfing is low. Specifically, *δσ, f*, *α _a_,* and *ι* **_A_** are *O*( *ξ*) and *β _a_, γ* **_A_** *, κ* **_A_** *_,a_,* and *κ* **_A_** *_,aa_* are *O*( *ξ* ^2^), where *ξ* ≪ 1. Under these assumptions, the change in the frequency of the *A* allele is



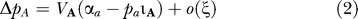



where *p _a_* is the frequency of allele *a* and *V* **_A_** = *p _A_p _a_* is the variance at locus **A**. (In general, the symbol *p _k_* is used to represent the frequency of allele *k,* and *V* **_K_** = *p _K_p _k_* is used to represent the variance at locus **K**.) Equation 2 shows that, to leading order, the rate of change of the *A* allele depends only on genotypic selection. The effects of maternal and similarity selection are negligible by comparison. This is not surprising as we assumed that these forms of selection are weak relative to genotypic selection.

Our primary interest is in the evolution of the modifier. Because the modifier has no direct effect on fitness, it evolves by developing associations with loci that are under selection. The leading order approximation for the change in the modifier is



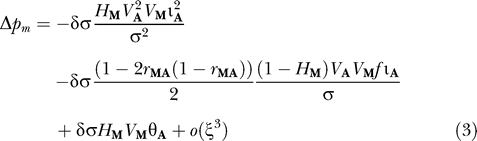



where *H* **_M_** = (1 − *h _σ_*) *p _m_* + *h _σ_p _M_* and *Θ* **_A_** = *V* **_A_**(2 *γ* **_A_** + (1 + 2 *p _a_*) *κ _A,a_* + *p _a_*κ _**A**, *aa*_).

The first two terms arise from genotypic selection alone and are similar to the results of [ 15]. The first term is always negative and reflects the fact that sex reduces genetic associations built by selection. The second term reflects selection on the modifier due to inbreeding. This term is slightly different from the analogous term in [ 15] because I have assumed sporophytic selfing rather than gametophytic selfing. The second term can be positive, favoring sex, if (1) inbreeding is present and (2) host–parasite interactions produce a negative value for *ι* **_A_**, as occurs with the Inverse Matching Alleles (IMA) model (see [ 11] for details). However, if there is very little inbreeding or *ι* **_A_** is positive, as occurs with the Matching Alleles (MA) model, then the sum of the first two terms will be negative, indicating that selection acts against sex.

A different picture emerges when we consider the third term, which arises from similarity selection. The factor *Θ* **_A_** represents the average increase in fitness of sexual offspring due to being different from their mothers at the **A** locus. This factor accounts for the fact that only some fraction of sexually produced offspring will be different from their mothers and that this fraction increases with allelic variance in the population. The sign of *Θ* **_A_** will depend on the nature of selection. Intuitively, *Θ* **_A_** is expected to be positive if hosts are biased towards encountering parasites from their mothers. Explicit host–parasite computer simulations described in Protocol S3 confirm this is the case (unpublished data).

Though the effects of similarity selection are negligible relative to the effects of genotypic selection with respect to the evolution of the fitness locus itself ( Equation 2), the effects of similarity selection are not negligible in shaping the evolution of the modifier. The third term in Equation 3 is of the same order as the first two terms despite the assumption that similarity selection is much weaker than genotypic selection. In other words, similarity selection is a much more potent force than genotypic selection in shaping the evolution of sex. If similarity selection is assumed to be *O*( *ξ*) rather than *O*( *ξ* ^2^), the terms involving genotypic selection drop out of the leading order approximation given in Equation 3. Consequently, the effects of similarity selection will dominate modifier evolution if similarity selection is only slightly weaker (as opposed to much weaker) than genotypic selection. Specifically, similarity selection drives modifier evolution when



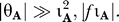



We can better understand these results by expressing Equation 3 in terms of the QLE associations:



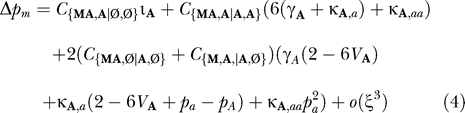



where *C*_{ ***O1***, ***O2|D1***, ***D2***}_ is the association among loci in set ***O1,O2*** in the offspring and the loci in set ***D1,D2*** in the dam. ***O1*** and ***D1*** refer to alleles at the first copy of each diploid locus, whereas ***O2*** and ***D2*** refer to alleles at the second copy. For example, *C*_{ **MA***,***A**|Ø,Ø}_ is an association involving loci only in offspring; specifically, it is the association between the modifier and homozygosity at the **A** locus in offspring. The other *C-*terms in the equation above refer to associations involving loci in both offspring and their dams. For example, *C*_{ **MA***,*Ø| **A**,Ø}_ is an association between the modifier and the similarity of allelic states between dam and offspring at the **A** locus. (All associations are measured as central moments of the frequency distribution following [ 20]. See Protocol S1 for details.)

By affecting reproduction, the modifier alters associations among combinations of alleles at other loci (e.g., *C*_{ **A***,***A**|Ø,Ø}_ and *C*_{ **A***,*Ø| **A***,*Ø} _). In the process, it becomes associated itself with those combinations of alleles (e.g., *C*_{ **MA***,***A**|Ø,Ø}_ and *C*_{ **MA***,*Ø| **A***,*Ø} _), with the magnitude of the latter associations being proportional to the former (e.g., *C*_{ **MA***,***A**|Ø,Ø}_ ∝ *C*_{ **A***,***A**|Ø,Ø} _, *C*_{ **MA***,*Ø| **A***,*Ø} _, and ∝ *C*_{ **A***,*Ø| **A***,*Ø} _). Evolution of the modifier occurs as a result of a correlated response to selection on those combinations of alleles at the **A** locus. Thus, the *potency* of similarity versus genotypic selection in affecting modifier evolution is mediated by the magnitude of the genetic associations upon which each form of selection acts.

Similarity selection acts on associations between alleles in offspring and alleles in their mothers (e.g., *C*_{ **A***,*Ø| **A***,*Ø} _). These associations tend to be large, *O*(1), because they are generated through inheritance. Similarity selection is assumed to be very weak *O*( *ξ* ^2^) but, because the associations it acts upon are large, the net contribution to change in the modifier's frequency is not too small, *O*( *ξ* ^3^). (An extra order of smallness arises because the modifier has only small effect on the amount of sex.)

Genotypic selection is assumed to be much stronger than similarity selection, though it is weak in absolute sense, *O*( *ξ*). Genotypic selection acts on associations between alleles within offspring (e.g., *C*_{ **A***,***A**|Ø,Ø} _). These associations tend to be weak, *O*( *ξ*), because they are built by forces that are assumed to be weak (i.e., non-linear genotypic selection, *ι* **_A_**, and/or inbreeding, *f*) relative to the mitigating effects of sex, *σ*. Consequently, the net contribution to change in the modifier's frequency arising from genotypic selection is also *O*( *ξ* ^3^).

Similarity selection is more potent than genotypic selection because it acts on genetic associations that are much larger than those acted upon by genotypic selection. This line of reasoning explains why similarity selection can be much weaker than genotypic selection but still be of equal importance with respect to the modifier's evolution. However, if the baseline level of sex is very low ( *σ* ≪ 1), associations between alleles within offspring (e.g., *C* **_A_** *_,_* **_A_**_|Ø,Ø_) can become stronger than *O*( *ξ*), thus increasing the potency of genotypic selection (see Equation 3).

### Explicit Consideration of Host–Parasite Coevolution

Intuition suggests that if hosts are sufficiently biased towards encountering parasites transmitted by their parents, host–parasite co-evolution should generate the type of similarity selection that favors sex. Explicit host–parasite models were used to examine this idea. (These models assume no selfing.) To allow for encounter bias, I assume there are two phases of transmission: maternal transmission is followed by global transmission. In the global transmission phase, a host encounters on average *λ* parasites, at random, from the global population of parasites. In the maternal transmission phase, a host encounters on average *φλ* parasites transmitted by their mothers. The parameter *φ* gives the ratio of maternal to global exposures; when *φ* ≪ 1, hosts primarily encounter parasites transmitted by unrelated individuals.

Though *φ* provides a convenient measure of the relative importance of maternal transmission, care is needed in interpreting the effects of changes in the parameter values. Increasing *λ*, while keeping *φ* constant, will increase both the number of global and maternal exposures. Under some conditions (e.g., decline in parasite density due to extrinsic factors), one might expect a decline in the number of global exposures but no change in the number of maternal exposures. Such a change would be represented by a reduction in *λ* accompanied by an increase in *φ*. Such environmental changes may often cause simultaneous changes in both parameters that are negatively correlated. However, the correlation between *λ* and *φ* will not be perfect, and when comparing parasite species with different life histories, the covariance between *λ* and *φ* is likely to low (i.e., these are not redundant parameters).

It is possible to calculate the parasite-induced selection on hosts in terms of the selection parameters used in the single-species model above. Table 2 shows these expressions for three different models of infection (see Protocol S2 for details). These analytical approximations rely on several simplifying assumptions that limit the parameter range over which they will be quantitatively accurate. Nonetheless, the expressions provide several key insights as to the qualitative effects different factors.

**Table 2:**
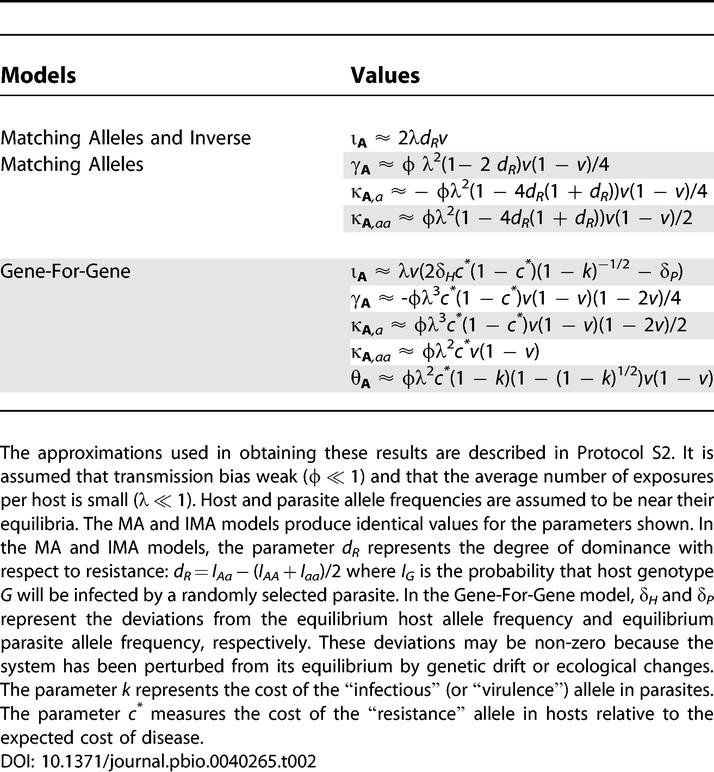
Relevant Parameter Values under Host–Parasite Coevolution

Assuming most encounters are random, i.e., *φ* ≪ 1, the strength of genotypic selection will depend on the probability that an individual becomes infected via a random encounter. This probability will be proportional to *λ,* provided that *λ* is not too large. The strength of similarity selection will depend on two probabilities: the probability that an individual's mother is infected *and* the probability that the individual is then infected by its mother. These two probabilities will be proportional to *λ* and *φλ*, respectively. Consequently, genotypic selection is proportional to *λ* whereas similarity selection depends on *φλ* ^2^. Thus, similarity selection will be weak relative to genotypic selection when most encounters are random ( *φ* ≪ 1). Holding *φ* constant, similarity selection is also expected to be weak if there are very few encounters of any kind ( *λ* ≪ 1). If encounters are rare, disease prevalence will be low amongst mothers, and only a small fraction of offspring will have the opportunity to be infected by their mothers, thus limiting the scope for similarity selection.

The second result evident from Table 2 is that genotypic and similarity selection are affected differently by the virulence of infection, *v*. Genotypic selection increases linearly with virulence. Similarity selection is maximized at intermediate virulence. If parasites are too virulent, infected mothers produce few offspring, and there is little opportunity in the population for transmission from mother to offspring. Similarity selection is strongest relative to genotypic selection when virulence is low.

**Figure 1:**
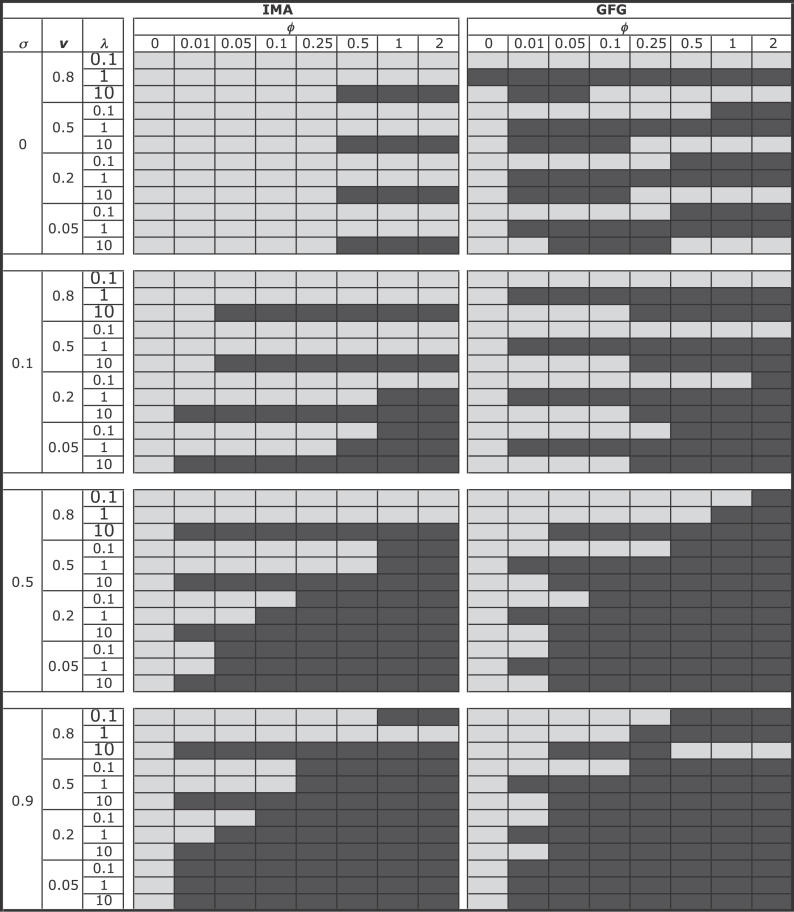
The Evolution of Sex with Exposure to Maternally Transmitted Parasites Darkly shaded regions indicate parameter values under which increased sex evolved in simulations. Lightly shaded regions indicate parameter values that selected against sex. A detailed description of the simulations is presented in Protocol S3. The effect of the modifier was assumed to be additive, i.e., *h _σ_* = ½. Left: two-locus IMA model with *d _R_* = 0.25. Right: two-locus Gene-For-Gene (GFG) model with a cost of resistance alleles of *c* = 0.05 *v* and cost of infectious alleles of *k* = 0.3. Resistance alleles were assumed to be completely dominant. MA model results were very similar to IMA model results (unpublished data). For the IMA model, the parameter combination with *σ* = 0.5, *v* = 0.2, *λ* = 1, and *φ* = 0.1, increased sex evolved in 90% of replicates; increased sex evolved in 95% of replicates the parameter combination with *σ* = 0.1, *v* = 0.8, *λ* = 10, and *φ* = 0.05; for other parameter combinations, sex evolved in all replicates in the direction indicated by shading.

Figure 1 shows the results of explicit host–parasite computer simulations described in Protocol S3. Unlike the analytical approximations that assume there is a single immunity locus, two loci mediate host–parasite interactions in the simulations reported here. In these simulations, parasites select against sex when encounters are random. In contrast, selection for sex often occurs if hosts are sufficiently biased towards encountering parasites produced by their mothers ( *φ >* 0). The extent of bias required to select for sex depends on various parameters that affect either the relative strength or potency of similarity versus genotypic selection.

Virulence, *v*, and the number of exposures, *λ*, affect the strength of similarity selection relative to genotypic selection. As described above, similarity selection is expected to be relatively stronger when virulence is low. Indeed, sex evolves more readily when virulence is low ( Figure 1). Sex also evolves more readily when the number of exposures, *λ*, is high. When the number of exposures is high, most individuals are infected regardless of genotype. Because genotype has only a small effect on probability of infection, selection on the genotype is weak, and the situation is analogous to low virulence from the perspective of the modifier. When the number of exposures is low, there is little opportunity for similarity selection because too few mothers are infected.

The baseline level of sex, *σ,* mediates the relative potency of genotypic selection. When the baseline level of sex is low, the associations between alleles within offspring (e.g., *C* **_A_** *_,_* **_A_**_|Ø,Ø_) become large (see Equation S1.6b in Protocol S1). Because these are the associations that are affected by genotypic selection ( Equation 4), the potency of genotypic selection is strong relative to similarity selection in these cases. Consequently, sex evolves less readily when the baseline level of sex, *σ,* is low.

It is worth noting that although parasites selected against increased sex in the simulations reported here when encounters were random, other studies [ 12, 13] have reported increases in genetic mixing with random encounters under some conditions (e.g., MA model with high virulence). Such studies have assumed hosts were haploid so that the effect of recombination on gametic disequilibrium was the only relevant factor to the evolution of sex. In the simulations presented here, hosts are diploid so that sex involves both the effect of segregation on homozgosity and the effect of recombination on gametic disequilibrium. As described in [ 11], the effects of segregation on homozgosity are expected to select against sex even under conditions when the effects of recombination on gametic disequilibrium favor sex. For example, in simulations of a two-locus diploid version of the MA model with high virulence, an allele that increased recombination increased in frequency, as expected from the haploid results, but an allele that increased investment into sexual reproduction declined in frequency (unpublished data). Thus, studies focusing on haploid hosts may have overestimated the potential for randomly encountered parasites to select for sex in diploids.

### Haploid Three-Locus Model

The Red Queen analysis by Otto and Nuismer [ 12] centered on a haploid three-locus model. For comparison, I present a haploid three-locus, single-species, model in which I allow the fitness of an individual to depend on the alleles it carries at two fitness loci ( **A** and **B**) as well as the extent to which its genotype is different from that of its mother (see Protocol S1 for details). With three di-allelic loci, there are eight genotypes. Because an individual's fitness depends on its own genotype as well as its mother's, it is necessary to keep track of the frequencies of 64 types of individuals (eight offspring genotypes from each of eight dam genotypes). The moments of the frequency distribution involve 57 association measures. In contrast, only four association measures are needed in the standard three-locus model.

As in the diploid model, a large number of selection parameters is needed to create a general fitness framework. The fitness function is constructed in a manner analogous to the diploid model. Fitness is defined by



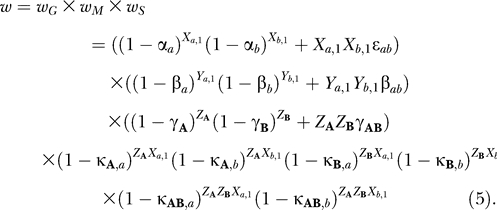



In this model, genotypic selection is described by *α _a_*, *α _b_*, and *ɛ _ab_*; maternal selection is described by *β _a_, β _b_,* and *β _ab_*; and similarity selection is described by *γ* **_A_**, *γ* **_B_** *, γ* **_AB_**, *κ* **_A_**_, *a*_ *, κ* **_A_**_, *b*_ *, κ* **_B_**_, *a*_ *, κ* **_B_**_, *b*_ *, κ* **_AB_**_, *a*_ *,* and *κ* **_AB_**_, *b*_ (see Table 1 for parameter definitions). Reproduction occurs following selection. Individuals with the *M* allele produce a fraction *σ* of their offspring sexually and the remainder asexually. Loci are in the order **MAB** with recombination rates *r* **_MA_** and *r* **_AB_** in the intervals **M** *-* **A** and **A** *-* **B**, respectively. In this model, the modifier allele, *m,* increases the allocation to sexual reproduction by an amount *δσ* and increases recombination in the intervals **M** *-* **A** and **A** *-* **B** by *δr* **_MA_** and *δr* **_AB_**, respectively. The analysis assumes *α _a_*, *α _b_*, *δσ*, *δr* **_MA_**, and *δr* **_AB_** are *O*( *ξ*), *ɛ _ab_* is *O*( *ξ* ^2^), and remaining selection coefficients, including all forms of similarity selection, are much weaker, *O*( *ξ* ^4^). At QLE, the change in the modifier is



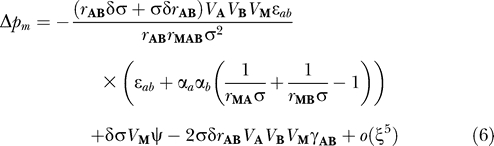



where *r* **_MAB_** = 1 − (1 − *r* **_MA_**)(1 − *r* **_AB_**) and *r* **_MB_** = *r* **_MAB_** − *r* **_MA_** *r* **_AB_** and *ψ =* 1/2 *V* **_A_**(2 *γ* **_A_** − 2 *γ* **_AB_** + *κ* **_A_** *_,a_ + p _B_*(2 *κ* **_A_** *_,b_* + *κ* **_A_** *_,ab_*)) + 1/2 *V* **_B_**(2 *γ* **_B_** − 2 *γ* **_AB_** + *κ* **_B_** *_,b_ + p _A_*(2 *κ* **_B_** *_,a_* + *κ* **_B_** *_,ab_*)) + 2 *V* **_A_** *V* **_B_**(1 − *r* **_AB_**) *γ* **_AB._**

The first term in Equation 6 is the typical result when an individual's fitness depends only on its own genotype (see [ 14] for the case when *σ* = 1 and *δσ* = 0; see [ 12] for the more general result). The remaining two terms arise because an individual's fitness depends on whether it shares alleles with its mother.

Two important points emerge from Equation 6. Otto and Nuismer [ 12] demonstrated that increases in sex ( *δσ* > 0) and increases in recombination ( *δr* > 0) are favored under exactly the same conditions when genotypic selection acts alone, as shown by the first term in Equation 6. However, sex and recombination are no longer selectively equivalent when similarity selection is included in the model. Similarity selection will favor sex whenever it is beneficial for an individual to have fewer alleles in common with its mother (second term in Equation 6). The conditions for similarity selection to favor recombination are more stringent; similarity selection only favors recombination when sharing alleles at two loci is worse than expected based on the fitness effects of sharing an allele at each locus alone (last term in Equation 6). If sharing alleles at two loci is deleterious but not as detrimental as expected based on single locus effects, then similarity selection acts against recombination even though it favors sex. The second important point from Equation 6 is that, as we saw in the previous model, similarity selection can be much weaker than genotypic selection but still be important in driving the evolution of sex and/or recombination. The results presented here with respect to recombination are not expected to apply to diploid models because recombination does not affect the variance in number of shared alleles between mother and offspring in diploids.

## Discussion

The idea that genetic mixing could be advantageous if individuals benefit from being different from their relatives is not new. However, hypotheses based on this idea have been largely abandoned for two reasons. First, these hypotheses were typically developed within the framework of obligately sexual groups competing against obligately asexual groups. Recently, there has been a shift away from models based on group-level explanations for genetic mixing [ 21, 22]. Second, similarity selection is generally thought to be much weaker than genotypic selection, and thus similarity selection is presumed to be unimportant [ 23]. However, a proper analysis of similarity selection using the modifier framework had been lacking.

Modifier models are extremely useful for identifying the conditions under which increases in genetic mixing can evolve within an interbreeding population. To date, such models have assumed that an individual's fitness depends only on its own genotype, i.e., genotypic selection. For the most part, these models have shown that it is difficult to increase the amount of genetic mixing in large, panmictic populations [ 14, 15]. Essentially, this is because genetic mixing usually reduces the genetic associations that are built by selection. Only if some additional force contributes to the development of disequilibrium (e.g., non-random mating or drift) does genetic mixing evolve under a broader range of selection parameters [ 24, 25].

Here I have presented modifier models that allow an individual's fitness to depend on its genotype as well as its similarity to its mother. The addition of similarity selection complicates the model because doing so requires tracking associations involving alleles in offspring with alleles in their mothers in addition to tracking genetic associations within offspring. However, such a model allows the effects of genotypic selection and similarity selection to be studied within a common framework for the first time. The results indicate that similarity selection is typically a much more potent force than genotypic selection. This increased potency occurs because similarity selection affects mother–offspring genetic associations that tend to be large, whereas genotypic selection acts on within-offspring genetic associations that tend to be weak. Consequently, similarity selection can have an equally large effect on the modifier's fate even if its effect on fitness is much weaker than that of genotypic selection.

Various ecological scenarios could generate similarity selection. One obvious source would be exposure of offspring to parasites transmitted by their mothers. Host–parasite coevolution has long been viewed as potentially playing an important role in the evolution of genetic mixing. In contrast to this idea, recent theory papers have argued that parasites tend to select against genetic mixing [ 11, 12, 26]. These models assumed that hosts encounter parasites completely at random, thereby excluding any possibility of similarity selection. I have found that by allowing for hosts to be biased towards encountering parasites from their mother, parasites are much more likely to favor sex. The extent of the exposure bias required to change the direction of selection on sex is quite low in some cases, though it is high in other cases. Notably, the gene-for-gene model of infection ( Figure 1), which has better empirical support than either of the “matching” models [ 27], favors sex with very low levels of exposure bias. Note that the models presented here do not include any intrinsic cost of sex because the primary question addressed is whether parasites create selection for sex or against it. (An intrinsic cost can easily be incorporated as in [ 11].) While it may be unlikely that selection by parasites can completely compensate for a two-fold intrinsic cost of sex, the results show that parasites often help to mitigate such a cost rather than add to it as previously suggested [ 11, 12, 26].

The model provides some interesting predictions on when parasites are most likely to favor sex. In systems in which offspring disperse widely before they can be exposed to parasites (or parasites disperse widely before having the opportunity to infect offspring), exposure bias will tend to be very low and of negligible importance. In such systems, parasites are unlikely to be selecting for sex by the mechanisms discussed here. At the other extreme, parasites are likely to be playing a more important role in systems in which parasites are directly vertically transmitted from mother to offspring. In previous models assuming random encounters, highly virulent parasites were sometimes capable of generating selection for sex [ 12, 13]. In contrast, the results presented here indicate that parasites with low virulence and/or high rates of infection are the most likely to favor sex. Similarity selection becomes weak if parasites are highly virulent or if infection rates are very low because, in either case, too few offspring are produced by infected mothers each generation. The results presented here also suggest that parasites are more effective at maintaining sex once it occurs at high levels rather than at its initial evolution. This is because genotypic selection, which typically acts against sex, is more potent when the baseline level of sex is low than when it is high.

The single-species analytical results (Equations 3 and 6) could, in principle, be applied to any type of species. Throughout I have assumed the focal species was the host, though these equations could also be applied to the parasite. For the same intuitive reason that exposure bias causes similarity selection favoring sex in hosts, exposure bias should cause similarity selection against sex in parasites. Though it is intriguing that many types of pathogens (e.g., viruses, bacteria, and fungi) are less sexual than their hosts, there are too many phylogenetically confounding variables to make this contrast very useful. A more interesting comparison would be to contrast levels of sexuality in parasitic taxa with their free-living sister taxa.

In the models presented, similarity selection depends only on the genetic similarity between offspring and mother. The results provide general insights into the effects of other types of similarity selection, which could be modeled using the same approach. For example, one could include fitness effects of the similarity between the genotypes of siblings. Such selection is the basis of the “tangled bank” hypothesis wherein individuals having a different genotype from their siblings enjoy reduced intra-family competition [ 7]. The effect on a modifier of this type of similarity selection should be analogous to that observed above. That is, sibling-based similarity selection is expected to be more potent than genotypic selection because the former affects larger genetic associations (associations among siblings) than does the latter (associations within individuals). In summary, with respect to loci that experience any type of similarity selection involving close relatives, this selection may be an important determinant of modifier evolution even if it is much weaker than genotypic selection.

## Supporting Information

Protocol S1 Deriving Analytical Approximations for Single-Species Modifier Models (440 KB PDF)

Protocol S2 Analytical Approximation of Selection Coefficients Generated by Host–Parasite Coevolution (610 KB PDF)

Protocol S3 Host–Parasite Simulations (441 KB PDF)

## Abbreviations

GFG: Gene-For-Gene
IMA: Inverse Matching Alleles
MA: Matching Alleles
QLE: quasi-linkage equilibrium
