# Supplementary material for: Similarity Selection and the Evolution of Sex: Revisiting the Red Queen
Source: PLoS Biol. 2006 Aug 1;4(8):e265. doi: 10.1371/journal.pbio.0040265 (PMC1523229; doi:10.1371/journal.pbio.0040265)
Supplement: Supplementary file 1 [file pbio.0040265.sd001.pdf]

Supporting Information 1. Deriving analytical approximations for single-species modifier models.

<u>Diploid, two-locus model</u>

As described in the main text, the **A** locus affects fitness and **M** locus affects an organisms investment into sexual versus asexual reproduction. There are two alleles at each locus resulting in four haplotypes *MA, Ma, mA,* and *ma*. Representing *M* and *A* alleles by 1 and *m* and *a* alleles by 0, these four haplotypes are denoted as {1,1}, {1,0}, {0,1}, and {0,0} respectively. A diploid genotype is the combination of two haplotypes. For example, the genotype *MA/ma* is {{1,1}, {0,0}}. The assignment of haplotype order is arbitrary, i.e., an individual consisting of one *MA* haplotype and one *ma* haplotype could be classified as either *MA/ma* or *ma/MA.* If an individual is classified as *ma/MA* then *ma* is considered its first haplotype and *MA* its second haplotype. An individual is described by the tensor $\boldsymbol{\Omega}$ that contains all relevant genetic information for determining its fitness, i.e., the individual's own genotype and its mother's genotype. $\boldsymbol{\Omega}$ represents the "extended genotype": an individual's own genotype as well the genotypes of any others that affect its fitness, which, in this case, is just the mother. Element $\Omega_{i,j,k}$ gives the allelic state of the individual at the kth locus ($k \in \{1,2\}$ for **M** and **A**, respectively) on the jth haplotype ($j \in \{1,2\}$ for the first and second haplotypes, respectively, that comprise the diploid) of the ith genome ($i \in \{1,2\}$ for individual's own genotype and its mother's genotype, respectively). For example, for an individual with genotype *MA/MA* from a mother with genotype *Ma/mA* the tensor $\boldsymbol{\Omega}$ would take the value {{{1,1}, {1,1}}, {{1,0}, {0,1}}. The frequency of individuals of type $\boldsymbol{\Omega}$ is given by $F_{\boldsymbol{\Omega}}$. Because the assignment of haplotype order is arbitrary, functionally equivalent types have equal frequency, e.g.,

$F_{\{\{\{1,1\},\{0,1\}\},\{\{1,1\},\{1,1\}\}\}} = F_{\{\{\{0,1\},\{1,1\}\},\{\{1,1\},\{1,1\}\}\}}$. Excluding equivalent types, it is necessary to track the frequencies of 100 different extended genotypes (10 non-equivalent offspring genotypes by 10 non-equivalent maternal genotypes).

As an alternative to describing the population by the extended genotype frequency distribution, we can use the allele frequency and the patterns of associations of alleles within the extended genotype. The frequency of the upper case allele (i.e., *M, A*) at the kth locus in the ith genome is given by $p_{i,k}$

$$p_{i,k} = \sum_{\Omega}\sum_{j} F_{\Omega}\Omega_{i,j,k} \qquad \text{(S1.1)}$$

For example, the frequency of the *M* allele among individuals of the current generation is $p_{1,1}$ whereas the frequency of *M* amongst the mothers of the individuals of the current generation is $p_{2,1}$. (For simplicity, in the main text $p_{1,1}$ is denoted simply as p_M and $p_{1,2}$ is denoted as p_A.) It is useful to note that in the absence of mutation, allele frequencies in the offspring at the beginning of a generation should equal the equivalent allele frequencies among their mothers, i.e., $p_{1,k} = p_{2,k}$. However, after selection this may no longer be the case.

In addition, to allele frequencies, it is necessary to quantify the associations among the allelic states in the extended genotype. The symbol $C_{\{\boldsymbol{O1},\boldsymbol{O2}|\boldsymbol{D1},\boldsymbol{D2}\}}$ represents the association among loci in set ***O1*,*O2*** in the offspring and the loci in set ***D1*,*D2*** in the dam. ***O1*** and ***D1*** refer to alleles in the first haplotype of offspring and maternal genomes, respectively, whereas ***O2*** and ***D2*** refer to alleles in the second haplotype of offspring and maternal genomes, respectively. For example, $C_{\{\mathbf{MA},\mathbf{A}|\varnothing,\varnothing\}}$ is an association involving loci

only in offspring; specifically, it is the three-way association among the modifier allele and the fitness allele on the first haplotype of the offspring genome and the fitness allele on the second haplotype of the offspring genome. (More simply, this association can be described as the association between the modifier and homozygosity at the **A** locus in offspring.) This association involves no loci in either haplotype comprising the maternal genome as signified by ∅,∅ in the subscript. In general, the association $C_{\{\boldsymbol{O1},\boldsymbol{O2}|\boldsymbol{D1},\boldsymbol{D2}\}}$ is quantified as

$$C_{\{\boldsymbol{O1},\boldsymbol{O2}|\boldsymbol{D1},\boldsymbol{D2}\}} = \sum_{\Omega}\left(F_{\Omega}\prod_{k1\in \boldsymbol{O1}}(\Omega_{1,1,k1}-p_{1,k1})\prod_{k2\in \boldsymbol{O2}}(\Omega_{1,2,k2}-p_{1,k2})\prod_{k3\in \boldsymbol{D}1}(\Omega_{2,1,k3}-p_{2,k3})\prod_{k4\in \boldsymbol{D}2}(\Omega_{2,2,k4}-p_{2,k4})\right) \tag{S1.2}$$

Products over empty sets are defined as 1; e.g., if $\boldsymbol{O1} = \{\varnothing\}$ then $\prod_{k\in \boldsymbol{O1}}(\Omega_{1,1,k}-p_{1,k})=1$. As shown by the equation above, associations are measured as central moments of the extended genotype frequency distribution. A complete description of the population requires that all possible associations are calculated (i.e., all subsets of $\{M,A\}$ in all possible combinations representing the four haplotypes of the extended genotype: ***O1***, ***O2***, ***D1***, and ***D2***). Because haplotype order is arbitrary, some association measures are redundant (e.g., $C_{\{\mathbf{M},\varnothing\,|\,\mathbf{A},\mathbf{M}\}} = C_{\{\varnothing,\mathbf{M}|\,\mathbf{A},\mathbf{M}\}} = C_{\{\mathbf{M},\varnothing\,|\,\mathbf{M},\mathbf{A}\}} = C_{\{\varnothing,\,\mathbf{M}|\,\mathbf{M},\mathbf{A}\}}$). Excluding redundant associations, there are 95 association measures needed to completely describe the population. There are 12 two-way associations (e.g., $C_{\{\mathbf{MA},\varnothing\,|\,\varnothing,\varnothing\}}$, $C_{\{\mathbf{A},\mathbf{A}\,|\,\varnothing,\varnothing\}}$, $C_{\{\mathbf{A},\varnothing\,|\,\mathbf{A},\varnothing\}}$, $C_{\{\varnothing,\varnothing\,|\,\mathbf{A},\mathbf{A}\}}$, etc.), 20 three-way associations, 26 four-way associations, 20 five-way

associations, 12 six-way associations, 4 seven-way associations, and 1 eight-way association.

Organisms are semelparous and the life cycle involves three stages: organisms are born, selection occurs, organisms reproduce. The pre-subscripts *b, s,* and *r* are added to the symbols above to denote these stages. For example, ${}_bF_{\boldsymbol{\Omega}}$ is the frequency of individuals with extended genotype $\boldsymbol{\Omega}$ before selection, ${}_sp_{1,1}$ is the frequency of M in individuals of the current generation after selection, and ${}_rC_{\{A,A\,|\,Ø,Ø\}}$ is a measure of homozygosity at the **A** locus in the offspring of the following generation. Note that values denoted as being "after reproduction" in one generation are equivalent to being "before selection" in the following generation, i.e., ${}_rF_{\boldsymbol{\Omega}}[t] = {}_bF_{\boldsymbol{\Omega}}[t + 1]$. In the main text, the pre-subscripts are omitted from these symbols for simplicity as all values presented there refer to values before selection.

The frequency of individuals with extended phenotype $\boldsymbol{\Omega}$ after selection is given by

$${}_sF_{\boldsymbol{\Omega}} = {}_bF_{\boldsymbol{\Omega}}w_{\boldsymbol{\Omega}}/E[w] \qquad \text{(S1.3)}$$

where $w_{\boldsymbol{\Omega}}$ is equation 1 from the main text evaluated with $X_{a,1} = 1 - \Omega_{1,1,2}$, $X_{a,2} = 1 - \Omega_{1,2,2}$, $Y_{a,1} = 1 - \Omega_{2,1,2}$, $Y_{a,2} = 1 - \Omega_{2,2,2}$, and

$$\bar{Z}_{\mathbf{A}} = \begin{cases} 1 \text{ for } \Omega_{1,1,2} + \Omega_{1,2,2} = \Omega_{2,1,2} + \Omega_{2,2,2} \\ 0 \text{ otherwise} \end{cases}$$

$E[w]$ is mean fitness, $E[w] = \Sigma_{\boldsymbol{\Omega}}({}_bF_{\boldsymbol{\Omega}}w_{\boldsymbol{\Omega}})$.

Reproduction follows selection. Considering the entire population, the fraction of offspring in the following generation produced sexually is $\sigma_T = \Sigma_{\boldsymbol{\Omega}}({}_sF_{\boldsymbol{\Omega}}\sigma_{\boldsymbol{\Omega}})$ where

$$\sigma_{\boldsymbol{\Omega}} = \begin{cases} \sigma \text{ if } \Omega_{1,1,1} + \Omega_{1,2,1} = 2 \\ \sigma + h_\sigma \delta\sigma \text{ if } \Omega_{1,1,1} + \Omega_{1,2,1} = 1 \\ \sigma + \delta\sigma \text{ if } \Omega_{1,1,1} + \Omega_{1,2,1} = 0 \end{cases}$$

The frequency of haplotype x amongst the gametes produced by an individual with extended genotype $\boldsymbol{\Omega}$ is $\Psi_{\boldsymbol{\Omega},x}$. These are haplotypes as normally defined (i.e., genotypes of the products of meiosis; they are not "extended haplotypes"). That is, $x \in \boldsymbol{S}$ where $\boldsymbol{S} = \{\{1,1\}, \{1,0\}, \{0,1\}, \{0,0\}\}$, i.e., the set of all possible haplotypes: *MA, Ma, mA,* and *ma*. The values for $\Psi_{\boldsymbol{\Omega},x}$ are calculated following the normal rules of meiosis. The total frequency of haplotype x in the sperm pool is

$$G_x = \sum_{\boldsymbol{\Omega}} {}_sF_{\boldsymbol{\Omega}} \frac{\sigma_{\boldsymbol{\Omega}}}{\sigma_T} \Psi_{\boldsymbol{\Omega},x} \qquad \text{(S1.4)}$$

Let the extended genotype $\{\{o1, o2\}, \{d1, d2\}\}$ represent an offspring carrying haplotypes $o1$ and $o2$ from a dam carrying haplotypes $d1$ and $d2$. The frequency of such offspring in the next generation is given by

$$
\begin{aligned}
{}_rF_{\{\{o1,o2\},\{d1,d2\}\}} &= \frac{1}{2}\sum_{x,y\in S} {}_sF_{\{\{d1,d2\},\{x,y\}\}}(1-\sigma_{\{\{d1,d2\},\{x,y\}\}})\chi_{\{o1,o2\},\{d1,d2\}} \\
&+\frac{1}{2}\sum_{x,y\in S} {}_sF_{\{\{d2,d1\},\{x,y\}\}}(1-\sigma_{\{\{d2,d1\},\{x,y\}\}})\chi_{\{o1,o2\},\{d2,d1\}} \\
&+\frac{1}{2}\sum_{x,y\in S} {}_sF_{\{\{d1,d2\},\{x,y\}\}}\sigma_{\{\{d1,d2\},\{x,y\}\}}\Psi_{\{\{d1,d2\},\{x,y\}\},o1}\left[f\Psi_{\{\{d1,d2\},\{x,y\}\},o2}+(1-f)G_{o2}\right] \\
&+\frac{1}{2}\sum_{x,y\in S} {}_sF_{\{\{d1,d2\},\{x,y\}\}}\sigma_{\{\{d1,d2\},\{x,y\}\}}\Psi_{\{\{d1,d2\},\{x,y\}\},o2}\left[f\Psi_{\{\{d1,d2\},\{x,y\}\},o1}+(1-f)G_{o1}\right]
\end{aligned}
\tag{S1.5}
$$

where $\chi_{\{x1,x2\},\{y1,y2\}}$ is an indicator variable that takes a value of one if $\{x1,x2\} = \{y1,y2\}$ (i.e., if the diploid genotypes are the same) but is otherwise zero. As defined in the main text, f is the fraction of sexually-produced offspring which are derived through sporophytic selfing. The first two terms in the equation above represent individuals created through asexual reproduction. There are two terms because the assignment of haplotype order is arbitrary. The latter two terms represent individuals created through sexual reproduction. Within the brackets of each of these latter terms, there are two terms representing individuals created through selfing and outcrossing, respectively.

Using the equations above, the extended genotype frequencies can be calculated at each stage of the life cycle. From these extended genotype frequencies, the allele frequencies and association measures can be calculated at each stage using equations S1.1 and S1.2. The goal is to determine the change in the frequency of the modifier over one complete generation, $\Delta p_M = {}_rp_{1,1} - {}_bp_{1,1}$. (In the main text, the change in the frequency of m, rather than M, is reported; the two are related by $\Delta p_m = -\Delta p_M$.) The equation for Δp_M is a complicated function that depends on various association measures. To make progress analytically, it is assumed that the association measures are close to their steady state values, i.e., the quasi-linkage equilibrium values, QLE; [1,2]. To

calculate the QLE values, the change in each association (e.g., $\Delta C_{\{O1,O2|D1,D2\}} = {}_{r}C_{\{O1,O2|D1,D2\}} - {}_{b}C_{\{O1,O2|D1,D2\}}$) is approximated by a Taylor series expansion under the assumptions of smallness described in the main text. After setting each of these equations for change to zero to represent the steady state, QLE values are obtained by simultaneously solving the system of equations for the associations. This is done by simultaneously solving for the 0th order approximation for all of the associations (which is zero for most associations). Using these 0th order approximations, the process is repeated to find first order approximations. The process is iterated until associations are known in sufficient detail. These approximate QLE values for the associations can then be used in calculating the change in the frequencies of *A* and *M* (e.g., equation 4 of main text). QLE values for the key associations driving the evolution of the modifier are as follows:

$$_{b(QLE)}C_{\{\{\mathbf{A},\varnothing|\,\mathbf{A},\varnothing\}\}} = \tfrac{1}{4}\, V_{\mathbf{A}}(2 - \sigma) + o(1) \qquad \text{(S1.6a)}$$

$$_{b(QLE)}C_{\{\{\mathbf{A},\mathbf{A}\,|\,\varnothing,\varnothing\}\}} = \tfrac{1}{2}\, V_{\mathbf{A}}(2V_{\mathbf{A}}(1 - \sigma)\iota_{\mathbf{A}} + f\sigma)/\sigma + o(\xi) \qquad \text{(S1.6b)}$$

$$_{b(QLE)}C_{\{\{\mathbf{MA},\mathbf{A}|\,\varnothing,\varnothing\}\}} = \delta\sigma V_{\mathbf{A}} V_{\mathbf{M}}\left(\frac{\iota_{\mathbf{A}} V_{\mathbf{A}} H_{\mathbf{M}}}{\sigma^2} + \frac{(1-2r_{\mathbf{MA}}(1-r_{\mathbf{MA}}))(1-H_{\mathbf{M}})f}{2\sigma}\right) + o(\xi^2) \qquad \text{(S1.6c)}$$

$$_{b(QLE)}C_{\{\{\mathbf{MA},\varnothing|\mathbf{A},\varnothing\}\}} = {}_{b(QLE)}C_{\{\{\mathbf{M},\mathbf{A}\,|\mathbf{A},\varnothing\}\}} = \tfrac{1}{4}\, \delta\sigma V_{\mathbf{A}} V_{\mathbf{M}} H_{\mathbf{M}} + o(\xi) \qquad \text{(S1.6d)}$$

$$_{b(QLE)}C_{\{\{\mathbf{MA},\mathbf{A}|\mathbf{A},\mathbf{A}\}\}} = \delta\sigma V_{\mathbf{A}}^2 V_{\mathbf{M}} H_{\mathbf{M}} + o(\xi) \qquad \text{(S1.6e)}$$

These QLE values are substituted into equation 4 of the main text to obtain equation 3 of the main text. (The pre-subscript "*b(QLE)*" is omitted in the main text for simplicity.)

To confirm the accuracy of the QLE approximation, simulations of the recursions above were compared to the QLE prediction. In these simulations, the *A* allele is beneficial and goes from low frequency to high frequency. At each generation during the sweep of *A*, the change in the frequency of the modifier allele *m* is calculated exactly from the simulation. The approximate change in the frequency of *m* is also calculated using the QLE prediction, i.e., equation 3 of the main text. In Figure S1.1, the actual changes are compared to the QLE predictions. The QLE prediction is usually in the same direction and is often close in magnitude to the actual change. As expected, the QLE prediction is least accurate when the baseline level of sex is low (e.g., $\sigma = 0.1$) and selection is strong.

<u>Haploid, three-locus model</u>

As described in the main text, the **A** and **B** loci affect fitness and **M** locus affects an organisms investment into sexual versus asexual reproduction as well as its recombination rates. The model follows the basic layout of the diploid, two-locus model.

As in the previous model, the extended genotype of an individual is described by the tensor $\boldsymbol{\Omega}$. Element $\Omega_{i,k}$ gives the allelic state of the individual at the kth locus ($k \in \{1,2,3\}$ for **M**, **A**, and **B**, respectively) of the ith genome ($i \in \{1,2\}$ for individual's own genotype and its mother's genotype, respectively). Because organisms are haploid, no index is needed to indicate first or second haplotype within a genome. Allele frequencies

and genetic associations are calculated as described above for the diploid model with the obvious adjustments for haploid organisms, i.e., sums and products are only done over one haplotype per genome rather than two. Symbols for the allele frequencies and associations are used analogously to the diploid model.

Selection is the first event in the life cycle. Extended genotype frequencies after selection are calculated following equation S1.3 where $w_{\boldsymbol{\Omega}}$ is equation 5 from the main text evaluated with $X_{a,1} = 1 - \Omega_{1,2}$, $X_{b,1} = 1 - \Omega_{1,3}$, $Y_{a,1} = 1 - \Omega_{2,2}$, $Y_{b,1} = 1 - \Omega_{2,3}$, $Z_{\mathbf{A}} = 1 - (\Omega_{1,2} - \Omega_{2,2})^2$, and $Z_{\mathbf{B}} = 1 - (\Omega_{1,3} - \Omega_{2,3})^2$.

Reproduction follows selection. The fraction of offspring in the following generation that will be produced sexually is $\sigma_T = \Sigma_{\boldsymbol{\Omega}}({}_sF_{\boldsymbol{\Omega}}\sigma_{\boldsymbol{\Omega}})$ where $\sigma_{\boldsymbol{\Omega}} = \sigma + \delta\sigma(1 - \Omega_{1,1})$. The frequency of extended genotype $\boldsymbol{\Omega}$ amongst the parents of offspring that will be produced by sex is ${}_sF_{\boldsymbol{\Omega}}\sigma_{\boldsymbol{\Omega}}/\sigma_T$. The frequency of haploid offspring of haplotype y amongst the progeny produced through sex between a female parent of extended genotype $\boldsymbol{\Omega 1}$ and a male parent of extended genotype $\boldsymbol{\Omega 2}$ is $\Psi_{\boldsymbol{\Omega 1}\times\boldsymbol{\Omega 2},y}$. Values for $\Psi_{\boldsymbol{\Omega 1}\times\boldsymbol{\Omega 2},y}$ are calculated following the rules of meiosis. The recombination rates used in employing these rules depend on the genotypes of the parents. The recombination rates in the **M**-**A** interval and the **A**-**B** interval are $r_{\mathbf{MA}} + \delta r_{\mathbf{MA}}(2 - (\Omega 1_{1,1} + \Omega 2_{1,1}))$ and $r_{\mathbf{AB}} + \delta r_{\mathbf{AB}}(2 - (\Omega 1_{1,1} + \Omega 2_{1,1}))$, respectively.

Let the extended genotype $\{o1, d1\}$ represent an offspring of haplotype $o1$ from a dam of haplotype $d1$. Considering both asexual and sexual reproduction, the frequency of such offspring in the next generation is given by

$$ {}_{r}F_{\{o1,d1\}} = \sum_{x \in \boldsymbol{S}} {}_{s}F_{\{d1,x\}}(1-\sigma_{\{d1,x\}})\chi_{o1,d1} + \sum_{x,y,z \in \boldsymbol{S}} {}_{s}F_{\{d1,x\}}\, {}_{s}F_{\{y,z\}}\sigma_{\{d1,x\}} \frac{\sigma_{\{y,z\}}}{\sigma_T} \Psi_{\{d1,x\}\times\{y,z\},o1} \qquad \text{(S1.7)} $$

This equation is analogous to equation S1.5. Here $\boldsymbol{S}$ = {{1,1,1}, {1,1,0}, {1,0,1}, {1,0,0}, {0,1,1}, {0,1,0}, {0,0,1}, {0,0,0}} to represent the haplotypes *MAB*, *MAb*, *MaB*, *Mab*, *mAB*, *mAb*, *maB*, and *mab*, respectively. Analogous to the diploid model, $\chi_{\{o1,d1\}}$ is an indicator variable that takes a value of one if $o1 = d1$ (i.e., if the haploid genotypes are the same) but is otherwise zero.

QLE values for the associations are calculated in a manner analogous to the diploid model. The QLE values for the key associations driving the evolution of the modifier in the haploid model are as follows:

$$ {}_{b(QLE)}C_{\{\mathbf{MA}|\varnothing\}} = V_{\mathbf{A}}V_{\mathbf{B}}V_{\mathbf{M}}\,(\delta\sigma\ r_{\mathbf{AB}} + \delta r_{\mathbf{AB}}\ \sigma)\alpha_b\varepsilon_{ab}/(\ r_{\mathbf{MA}}\ r_{\mathbf{AB}}\ r_{\mathbf{MAB}}\ \sigma^3) + o(\xi^4) \qquad \text{(S1.8a)} $$

$$ {}_{b(QLE)}C_{\{\mathbf{MB}|\varnothing\}} = V_{\mathbf{A}}V_{\mathbf{B}}V_{\mathbf{M}}\,(\delta\sigma\ r_{\mathbf{AB}} + \delta r_{\mathbf{AB}}\ \sigma)\alpha_a\varepsilon_{ab}/(\ r_{\mathbf{MB}}\ r_{\mathbf{AB}}\ r_{\mathbf{MAB}}\ \sigma^3) + o(\xi^4) \qquad \text{(S1.8b)} $$

$$ {}_{b(QLE)}C_{\{\mathbf{MAB}|\varnothing\}} = V_{\mathbf{A}}V_{\mathbf{B}}V_{\mathbf{M}}\,(\delta\sigma\ r_{\mathbf{AB}} + \delta r_{\mathbf{AB}}\ \sigma)\varepsilon_{ab}/(r_{\mathbf{AB}}\ r_{\mathbf{MAB}}\ \sigma^2) + o(\xi^3) \qquad \text{(S1.8c)} $$

$$ {}_{b(QLE)}C_{\{\mathbf{MA}|\mathbf{A}\}} = \tfrac{1}{2}\ V_{\mathbf{A}}V_{\mathbf{M}}\ \delta\sigma\ + o(\xi) \qquad \text{(S1.8d)} $$

$$ {}_{b(QLE)}C_{\{\mathbf{MB}|\mathbf{B}\}} = \tfrac{1}{2}\ V_{\mathbf{B}}V_{\mathbf{M}}\ \delta\sigma\ + o(\xi) \qquad \text{(S1.8e)} $$

$$ {}_{b(QLE)}C_{\{\mathbf{MAB}|\mathbf{AB}\}} = \tfrac{1}{2}\ V_{\mathbf{A}}V_{\mathbf{B}}V_{\mathbf{M}}\,(\delta\sigma(1 + r_{\mathbf{AB}}) + \delta r_{\mathbf{AB}}\ \sigma) + o(\xi) \qquad \text{(S1.8f)} $$

where $r_{\mathbf{MAB}} = 1 - (1 - r_{\mathbf{MA}})\ (1 - r_{\mathbf{AB}})$ and $r_{\mathbf{MB}} = r_{\mathbf{MAB}} - r_{\mathbf{MA}}\ r_{\mathbf{AB}}$. Using the QLE approximations above, the change in the modifier is found to be

$$ \Delta p_m = {}_{b(QLE)}C_{\{\mathbf{MA}|\varnothing\}}\alpha_a + {}_{b(QLE)}C_{\{\mathbf{MB}|\varnothing\}}\alpha_b - {}_{b(QLE)}C_{\{\mathbf{MAB}|\varnothing\}}(\alpha_a\alpha_b + \varepsilon_{ab}) $$

$$+ {}_{b(QLE)}C_{\{\mathbf{MA|A}\}}(2\gamma_{\mathbf{A}} - 2\,\gamma_{\mathbf{AB}}(1 - 2\mathrm{V}_{\mathbf{B}}) + \kappa_{\mathbf{A},a} + p_B(2\kappa_{\mathbf{A},b} + \kappa_{\mathbf{A},ab}))$$

$$+ {}_{b(QLE)}C_{\{\mathbf{MA|A}\}}(2\gamma_{\mathbf{B}} - 2\,\gamma_{\mathbf{AB}}(1 - 2\mathrm{V}_{\mathbf{A}}) + \kappa_{\mathbf{B},a} + p_A(2\kappa_{\mathbf{A},b} + \kappa_{\mathbf{A},ab}))$$

$$- 4\,{}_{b(QLE)}C_{\{\mathbf{MAB|AB}\}}\gamma_{\mathbf{AB}} + o(\xi^5) \qquad \text{(S1.9)}$$

Substitution of equations S1.8a-f into the equation above gives equation 6 of the main text.

Extension to other studies of similarity selection

In principle, the methods described here could be used to investigate other models incorporating similarity selection. A key step is defining the extended genotype appropriately to contain all necessary information. This means that the extended genotype must include information on all other individuals who affect the fitness of the focal individual. For example, in the models above, the extended genotype contained information an individual's own genotype as well as its mother's genotype. If one was investigating a model in which an individual's fitness depended on its siblings (e.g., Tangled Bank), then the extended genotype would need to include information on the genotypes of siblings. In practice, it would be become very difficult to use extended genotypes containing individual genotypes for more than a few siblings. One potential solution would be to use summary statistics to describe the genetic properties of the relevant relatives. For example, rather than listing the genotypes of each sibling as part of the extended genotype, one could simply include only the mean allele frequencies among siblings. It is necessary that fitness be defined in a compatible manner. For example, if the extended genotype included information only on the mean allele

frequencies among siblings, then the fitness function must depend only on the genotype of the individual and mean allele frequencies of its siblings.

Literature Cited

1. Kimura M (1965) Attainment of quasi linkage equilibrium when gene frequencies are changing by natural selection. Genetics 52: 875-&.
2. Barton NH, Turelli M (1991) Natural and sexual selection on many loci. Genetics 127: 229-255.

Figure S1.1. Comparison of QLE prediction to actual changes in modifier frequency. Simulations were performed using the exact recursions for the single-species diploid model. Plots show the change in the frequency of the *m* allele each generation. The black line shows the exact changes in p_m; the grey line shows the changes predicted by the QLE approximation (equation 3 of main text) using the actual allele frequencies at the beginning of each generation. The top, middle, and bottom rows show results for simulations with low ($\sigma = 0.1$), intermediate ($\sigma = 0.5$), and high ($\sigma = 0.9$) baseline levels of sex, respectively. Values for the selection parameters α_a, $\iota_{\mathbf{A}}$, and $\gamma_{\mathbf{A}}$ are given above each column. In all simulations shown, the following parameter values were used: $\beta_a = \kappa_{\mathbf{A},a} = \kappa_{\mathbf{A},aa} = f = 0$, $r_{\mathbf{MA}} = 0.2$, $\delta\sigma = 0.01$, $h_\sigma = 0.5$.

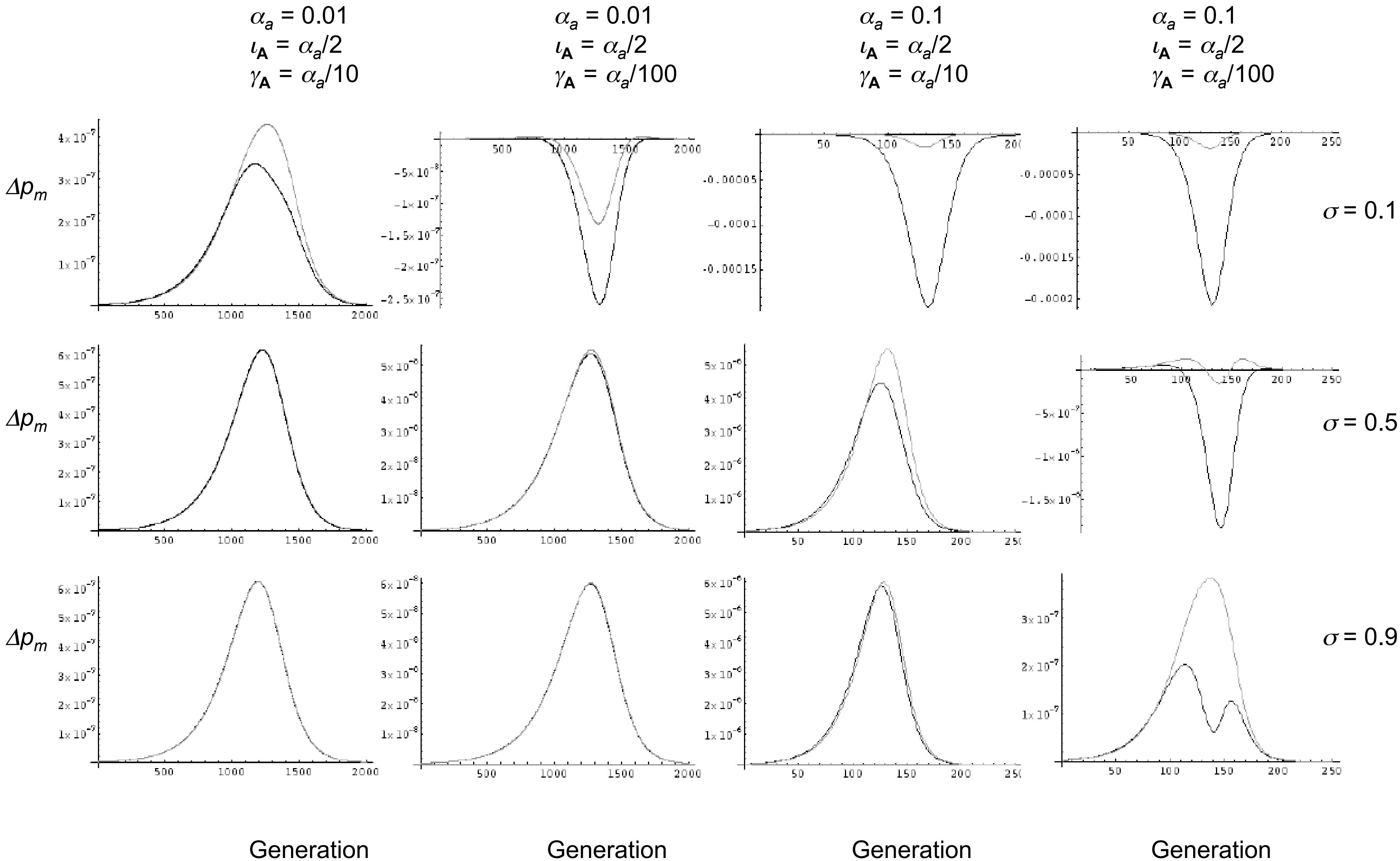

α_a = 0.01
ι_A = α_a/2
γ_A = α_a/10
α_a = 0.01
ι_A = α_a/2
γ_A = α_a/100
α_a = 0.1
ι_A = α_a/2
γ_A = α_a/10
α_a = 0.1
ι_A = α_a/2
γ_A = α_a/100
Δp_m
σ = 0.1
Δp_m
σ = 0.5
Δp_m
σ = 0.9
Generation
Generation
Generation
Generation
