# Supplementary material for: Similarity Selection and the Evolution of Sex: Revisiting the Red Queen
Source: PLoS Biol. 2006 Aug 1;4(8):e265. doi: 10.1371/journal.pbio.0040265 (PMC1523229; doi:10.1371/journal.pbio.0040265)
Supplement: Supplementary file 2 [file pbio.0040265.sd002.pdf]

Supporting Information 2. Analytical approximation of selection coefficients generated by host-parasite coevolution.

Intuitively, host-parasite coevolution in which offspring are biased towards encountering parasites transmitted by their own mothers should result in both genotypic and similarity selection. Here I describe how to calculate the selection generated in an one-locus host-parasite model in terms of the selection coefficients used in the single-species model presented in the main text (i.e., equation 1).

First, I determine the relationship between the selection coefficients and the fitnesses of different offspring/mother combinations. Let $w_{i,j}$ be the fitness of an offspring of genotype i from a mother of genotype j. Using the single-species fitness function given by equation 1, multiplied by a scaling factor K, we obtain a set of equations relating fitnesses to selection coefficients: $w_{AA,AA} = K(1 - \gamma_{\mathbf{A}})$, $w_{AA,Aa} = K(1 - \beta_a)$, $w_{Aa,AA} = K(1 - \alpha_a)$, $w_{Aa,Aa} = K(1 - \alpha_a)(1 - \beta_a)(1 - \gamma_{\mathbf{A}})(1 - \kappa_{\mathbf{A},a})$, $w_{Aa,aa} = K(1 - \alpha_a)(1 - \beta_a)^2(1 - \gamma_{\mathbf{A}})(1 - \kappa_{\mathbf{A},a})$, $w_{aa,Aa} = K((1 - \alpha_a)^2 + \iota_{\mathbf{A}})(1 - \beta_a)$, and $w_{aa,aa} = K((1 - \alpha_a)^2 + \iota_{\mathbf{A}})(1 - \beta_a)^2(1 - \gamma_{\mathbf{A}})(1 - \kappa_{\mathbf{A},a})^2(1 - \kappa_{\mathbf{A},aa})$. Rearranging these equations, the selection parameters can be calculated as:

$$K = \frac{w_{AA,Aa}\sqrt{w_{Aa,AA}}}{\sqrt{w_{Aa,aa}}} \quad \text{(S2.1)}$$

$$\alpha_a = 1 - \frac{\sqrt{w_{Aa,AA}w_{Aa,aa}}}{w_{AA,Aa}} \quad \text{(S2.2)}$$

$$\iota_{\mathbf{A}} = \frac{w_{AA,Aa} w_{aa,Aa} - w_{Aa,AA} w_{Aa,aa}}{w_{AA,Aa}^2} \tag{S2.3}$$

$$\beta_a = 1 - \sqrt{\frac{w_{Aa,aa}}{w_{Aa,AA}}} \tag{S2.4}$$

$$\gamma_{\mathbf{A}} = 1 - \frac{w_{AA,AA} \sqrt{w_{Aa,aa}}}{w_{AA,Aa} \sqrt{w_{Aa,AA}}} \tag{S2.5}$$

$$\kappa_{\mathbf{A},a} = 1 - \frac{w_{AA,Aa} w_{Aa,Aa}}{w_{AA,AA} w_{Aa,aa}} \tag{S2.6}$$

$$\kappa_{\mathbf{A},aa} = 1 - \frac{w_{AA,AA} w_{Aa,AA} w_{Aa,aa} w_{aa,aa}}{w_{AA,Aa} w_{Aa,Aa}^2 w_{aa,Aa}} \tag{S2.7}$$

To make use of these results, we must determine the fitnesses of each offspring/mother combination in the host-parasite model. Below I describe the host-parasite model and then how to determine the fitnesses of each offspring/mother combination.

Hosts are assumed to be diploid and capable of both sexual and asexual reproduction; parasites are assumed to be haploid and obligately asexual. Each generation begins with the birth of host offspring. Offspring born to infected mothers are exposed to parasites produced by their own mothers. During this maternal infection phase, these offspring are exposed to $\phi\lambda$ parasites, on average. All offspring then experience a global infection phase during which they are exposed to the global pool of parasite genotypes in the population. On average, hosts encounter λ parasites during this

infection phase. The parameter ϕ is a measure of the bias towards encountering parasites transmitted by one's own mother. The values $\phi\lambda$ and λ, representing maternal and global exposures, should be interpreted as the average number of "effective" encounters, i.e., encounters in which the probability that the host becomes infected is determined by the compatibility of the interaction as determined by the loci of interest (in this case, locus **A**). General mechanisms of resistance (e.g., behavioural avoidance of infected food items, thick skin preventing parasite penetration) should be thought of as factors that reduce the effective number of encounters. This exposure process is modeled by assuming that there are τ independent episodes in which a host might experience an encounter with a parasite during each of the two transmission phases ($\tau > \phi\lambda, \lambda$). The probability that a host has an *effective* encounter with a parasite during an episode is $b_H = \lambda/\tau$ for the global infection phase and $b_V = \phi\lambda/\tau$ for the maternal infection phase.

The probability that an encounter between an uninfected offspring of genotype g and a parasite of genotype j will result in an infection is given by $I_{g,j}$. In an effective encounter, the susceptibility of an individual host to a particular parasite is determined by each individual's genotype at the **A** locus and the model of infection used (e.g., MA, IMA, GFG – see Tables S2.1-S2.3). Though hosts may encounter multiple parasites, each host can only be infected by a single parasite.

As the fitness of an offspring will depend on whether its mother is infected or not, we must first calculate infection frequencies among mothers of each genotype. To do so, I assume that the exposure bias is small ($\phi << 1$), i.e., only a very small fraction of exposures to parasites involve parasites transmitted by a host's own mother. With this assumption, we can approximate the fraction of mothers in each infection state by

considering only the global infection phase. From this we can calculate the frequency of each offspring genotype produced by mothers of each genotype in each infection state. Allowing for both maternal and global transmission, it is possible to calculate the fitness of a specific offspring genotype when produced by a mother in a specific infection state. By taking a weighted average over each of the possible infection states of the mother, the expected fitness of an offspring of genotype g from a mother of genotype h can be calculated, i.e., $w_{g,h}$. Below I describe these steps in detail.

In order to calculate the fitness of each mother-offspring genotype combination, we need to know the frequency of mothers of each genotype in each infection state. If most infections occur via global transmission (i.e., ϕ <<1), then the infection frequency of mothers can be approximated by considering only this transmission phase. The probability that an uninfected individual of genotype g remains uninfected after a single encounter from with a globally transmitted parasite is

$$U_{H,g} = (1 - b_H) + b_H(q_A(1 - I_{g,A}) + q_a(1 - I_{g,A})) \qquad \text{(S2.8)}$$

where q_i is the frequency of parasite genotype i. As we are determining the frequency of infection amongst mothers, here the q_i refer to parasite frequencies during the previous (i.e., maternal) generation. Considering all encounters during the global transmission phase, the frequency of uninfected individuals of genotype g is

$$F_{I,g,\varnothing} = F_g U_{H,g}^{\tau} \qquad \text{(S2.9)}$$

where F_g is the initial of individuals of genotype g in the previous (i.e., maternal) generation. The frequency of individuals of genotype g that are infected by parasite i is given by

$$F_{I,g,i} = F_g b_H q_i I_{g,i} \sum_{k=1}^{\tau} U_{H,g}^{k-1} = F_g b_H q_i I_{g,i} \frac{1-U_{H,g}^{\tau}}{1-U_{H,g}} \qquad \text{(S2.10)}$$

Let $w_{H,g,j}$ be the fitness of a host of genotype g in infection state $j \in \{A, a, \varnothing\}$ (representing infection by parasite A, infection by parasite $a,$ and uninfected, respectively). For MA and IMA models, uninfected individuals have a fitness of 1, i.e $w_{H,g,\varnothing} = 1$. For the GFG model, $w_{H,aa,\varnothing} = 1$ and $w_{H,AA,\varnothing} = w_{H,Aa,\varnothing} = 1 - c$ where c is the cost of carrying at least one copy of the resistance allele. Following the empirical literature, the resistant allele A is assumed to be completely dominant to a. (The approximations presented in Table 2 of the main text are expressed in terms of the cost of resistance relative to the expected cost of infection, $c^* = c/\lambda v$.) Infection reduces host fitness by a factor v, relative to the uninfected state so that $w_{H,g,j} = (1-v) w_{H,g,\varnothing}$ for $j \neq \varnothing$. The mean fitness of in the maternal generation is given by

$$\overline{w}_H = \sum_j F_{I,g,j} w_{H,g,j} \qquad \text{(S2.11)}$$

The contribution of mothers with genotype g in infection state j to the following generation is

$$C_{g,j} = F_{I,g,j} w_{H,g,j} / \overline{w}_H \tag{S2.12}$$

The frequency of the A allele in the gamete pool is

$$G_A = C_{AA,A} + C_{AA,a} + C_{AA,\varnothing} + (C_{Aa,A} + C_{Aa,a} + C_{Aa,\varnothing})/2 \tag{S2.13}$$

and the frequency of the alternative allele is $G_a = 1 - G_A$. The frequency of offspring of genotype g produced by mothers of genotype h in infection state i is

$$F'_{M,g,h,i} = C_{h,i}\left(B_{g,h}(1-\sigma) + \sigma \sum_x \sum_y \Psi_{h,x} G_y K_{g,x,y}\right) \tag{S2.14}$$

where σ is the fraction of offspring produced sexually, the remainder being produced asexually. (All individuals are assumed to invest equally into sexual reproduction, which is a good approximation provided the effects of sex modifiers are small.) $B_{g,h}$ is an indicator variable that is 1 if $g = h$ but is zero otherwise. $\Psi_{h,x}$ is the frequency of haplotype $x \in \{A, a\}$ amongst the gametes produced by genotype h (e.g., $\Psi_{AA,A} = 1$, $\Psi_{Aa,A} = ½$). $K_{g,x,y}$ is an indicator variable that takes a value of one if the combination of haplotype x and haplotype y create a diploid of genotype g but is otherwise zero (e.g., (e.g., $K_{AA,A,A} = 1$, $K_{Aa,A,A} = 0$). The prime in $F'_{M,g,h,i}$ denotes that the symbol refers to the offspring generation rather than the maternal generation. (Note that the quantity used in $F'_{M,g,i}$ used in S3 is simply $\sum_h F'_{M,g,h,i}$.)

Only parasites that successfully infect a host contribute to the next generation. In the MA and IMA models, parasites that have successfully infected a host have a fitness of 1; unsuccessful parasites have a fitness of 0. In the GFG model, there is a cost to the 'infectious' allele a in parasites. Parasites of genotype A that successfully infect a host have a fitness of 1 whereas successful parasites of genotype a have a fitness of $1 - k$. Using these rules it is trivial to calculate the global parasite frequencies, $q_A^{'}$ and $q_a^{'}$, in the following generation.

Because of the possibility of maternal transmission, the fitness of offspring will depend on the infection state of their mothers. The probability that an uninfected offspring of genotype g from a mother in infection state j remains uninfected after a single encounter with a maternally transmitted parasite is

$$U_{V,g,j}^{'} = (1 - b_V) + b_V(1 - I_{g,j}) \qquad \text{(S2.15)}$$

(In the case of offspring from uninfected mothers, we define $I_{g,\varnothing} = 0$ so that $U_{V,g,\varnothing}^{'} = 1$.) The probability that an uninfected offspring of genotype g remains uninfected after a single encounter with a globally transmitted parasite is

$$U_{H,g}^{'} = (1 - b_H) + b_H(q_A^{'}(1 - I_{g,A}) + q_a^{'}(1 - I_{g,a})) \qquad \text{(S2.16)}$$

Considering both transmission phases, the probability that an offspring of genotype g from a mother in infection state i is in infection state j is

$$P'_{I,g,i,j} = \begin{cases} b_V f_{V,i,j} I_{g,j} \dfrac{1-(U'_{V,g,i})^{\tau}}{1-U'_{V,g,i}} + b_H q'_j I_{g,j} (U'_{V,g,i})^{\tau} \dfrac{1-(U'_{H,g})^{\tau}}{1-U'_{H,g}} & \text{for } i \neq \varnothing \text{ and } j \neq \varnothing \\ b_H q'_j I_{g,j} \dfrac{1-(U'_{H,g})^{\tau}}{1-U'_{H,g}} & \text{for } i = \varnothing \text{ and } j \neq \varnothing \\ (U'_{V,g,i} U'_{H,g})^{\tau} & \text{for } i \neq \varnothing \text{ and } j = \varnothing \\ (U'_{H,g})^{\tau} & \text{for } i = \varnothing \text{ and } j = \varnothing \end{cases}$$

(S2.17)

where $f_{V,i,j}$ is 1 if $i = j$ and is 0 otherwise because it is assumed parasites reproduce asexually and without mutation.

The average fitness of an offspring of genotype g from a mother of genotype h can be calculated as

$$w_{g,h} = \frac{\sum_i \sum_j F'_{M,g,h,i} P'_{I,g,i,j} w_{H,g,j}}{\sum_i F'_{M,g,h,i}} \qquad \text{(S2.18)}$$

The single species selection coefficients can be calculated by substituting these values into equations S2.1-7.

To obtain interpretable analytical approximations for these selection coefficients (Table 2) a number of assumptions are made. As stated previously, the exposure bias is assumed to be small. Technically, ϕ is $O(\xi)$ where $\xi << 1$. I also assume that the number of exposures is small, allele frequencies in the parental generation of hosts and parasites are close to equilibrium and that the parental generation is close to Hardy-Weinberg before selection. Specifically, the average number of exposures is $\lambda = b\tau$ (see Supporting

Information 3) where b is $O(\xi^2)$ and τ is $O(1/\xi)$ so that λ is $O(\xi)$. The initial genotype frequencies in the parental generation of hosts is $H_{AA} = (p_A^* + \delta_H) + C_{A,A}$, $H_{Aa} = 2(p_A^* + \delta_H)(p_a^* - \delta_H) - 2C_{A,A}$, and $H_{aa} = (p_a^* - \delta_H)^2 + C_{A,A}$, where p_i^* is the equilibrium frequency of allele i, δ_H represents the deviation from equilibrium allele frequency and $C_{A,A}$ is a measure of homozygosity; δ_H and $C_{A,A}$ are both assumed to be small, $O(\xi)$. The initial frequencies of parasites in the parental generation are $P_A = q_A^* + \delta_P$ and $P_a = 1 - (q_A^* + \delta_P)$ where q_A^* is the equilibrium frequency of A in parasites and δ_P represents the deviation from equilibrium parasite allele frequency; δ_P is $O(\xi)$. The deviations from equilibrium allele frequencies, δ_H and δ_P, may be non-zero because the population has not yet reached equilibrium or has been perturbed from equilibrium from some force such as drift or an ecological distrubance. In the MA and IMA models, $p_A^* = q_A^* = ½$. In the GFG model,

$$p_A^* \approx 1 - \sqrt{1-k}\left(1 - \lambda c^* k/4\right) \tag{S2.19}$$

and

$$q_A^* \approx c^*(1 + \lambda(1-v)) - \lambda(c^*)^2(1-2v)/2 \tag{S2.20}$$

In calculating this GFG equilibrium, the cost of resistance is assumed to be small, $c << 1$. (Note that if c is too high then resistance alleles are eliminated from the population.). Using these equilibrium values we calculate the selection coefficients using equations S1.1 - S1.7 to obtain the results presented in Table 2.

Supporting Information 2 - Tables

	Parasite Genotype	
Host Genotype	*A*	*a*
AA	1	0
Aa	½ + d_R	½ + d_R
aa	0	1

Table S2.1. Probability of infection of host upon exposure to parasite. One-locus Matching Alleles (MA) model.

	Parasite Genotype	
Host Genotype	*A*	*a*
AA	0	1
Aa	½ + d_R	½ + d_R
aa	1	0

Table S2.2. Probability of infection of host upon exposure to parasite. One-locus Inverse Matching Alleles (IMA) model. Note that the one-locus version of the IMA model is essentially the same as the MA model though parasite alleles are labeled differently. The biological motivation behind the IMA model would suggest a negative value for d_R whereas a positive value would be expected for the MA model. (The two-locus versions of the IMA and MA models described in S3 are not equivalent.)

	Parasite Genotype	
Host Genotype	*A*	*a*
AA	0	1
Aa	0	1
aa	1	1

Table S2.3. Probability of infection of host upon exposure to parasite. One-locus Gene-For-Gene (GFG) model.
