# Supplementary material for: Similarity Selection and the Evolution of Sex: Revisiting the Red Queen
Source: PLoS Biol. 2006 Aug 1;4(8):e265. doi: 10.1371/journal.pbio.0040265 (PMC1523229; doi:10.1371/journal.pbio.0040265)
Supplement: Supplementary file 3 [file pbio.0040265.sd003.pdf]

Supporting Information 3. Host-parasite simulations

Deterministic computer simulations were performed to evaluate the effect of maternally-transmitted parasites on the evolution of sex. Briefly, the simulations work as follows. Hosts are assumed to be diploid and parasites to be haploid. Both species have non-overlapping generations. Each generation begins with the birth of host offspring. If they are produced by infected mothers, offspring are exposed to parasites transmitted by their mother (maternal infection phase). All offspring, regardless of maternal infection status, experience a global transmission phase in which they are exposed to parasites transmitted by any infected host of parental generation. Reproduction follows these transmission phases. Hosts produce some fraction of their offspring sexually and the remainder asexually. An individual's investment into sex is determined by its genotype at the **M** locus. Infected individuals produce less offspring than uninfected individuals. Generations are non-overlapping, i.e., mothers die immediately following production of offspring and transmission of parasites. A more detailed description follows.

Let n_H and n_P be the number of host and parasite genotypes respectively. Let $n_I = n_P + 1$ be the number of possible infection states for a host; a host can be infected by one of the n_P parasite genotypes or be uninfected. Let $F_{M,g,i}$ be the frequency of offspring in the population that are of genotype g from mothers in infection state i. For $1 \le i \le n_P$, infection state i means that the host is infected by parasite genotype i. Infection state $i = n_P + 1$ corresponds to being uninfected.

There are two infection phases: the maternal infection phase followed by the global infection phase; offspring from uninfected mothers only experience the latter. During the global infection phase, a host encounters, on average, λ parasites. During the

maternal infection phase, a host encounters $\phi\lambda$ parasites, on average. (The parameter ϕ represents the ratio of maternal exposures to global exposures.) This is modeled by assuming that there are τ independent episodes in which a host might encounter a parasite during each of the two transmission phases ($\tau > \phi\lambda, \lambda$). The probability that a host actually encounters a parasite during an episode is $b_H = \lambda/\tau$ for the global infection phase and $b_V = \phi\lambda/\tau$ for the maternal infection phase. Note that modeling encounters in this manner is an approximation to a Poisson distribution; as $\tau \rightarrow \infty$ the distribution of number of encounters becomes Poisson with means of $\phi\lambda$ and λ for the maternal and global phases, respectively. If a host does encounter a parasite during an episode, it may or may not become infected, depending on the compatibility of the interaction. The probability that an encounter between an uninfected offspring of genotype g and a parasite of genotype j will result in an infection is given by $I_{g,j}$ (see Tables S3.1-S3.3). Once a host becomes infected, it cannot be infected by additional parasites.

Let $U_{V,g,i}$ be the probability that an uninfected offspring of genotype g from a mother in infection state i remains uninfected after a single encounter from with a maternally transmitted parasite:

$$U_{V,g,i} = \begin{cases} (1-b_V) + b_V \sum_{j}^{n_P} f_{V,i,j}(1-I_{g,j}) & \text{for } i < n_P + 1 \\ 1 & \text{for } i = n_P + 1 \end{cases} \quad \text{(S3.1)}$$

where $f_{V,i,j}$ is the frequency of parasites of genotype j among the parasites produced by a mother in infection state i (i.e., this is the frequency of parasite j among maternally transmitted parasites from a mother in state i). Let $U_{H,g}$ be the probability that an

uninfected offspring of genotype g remains uninfected after a single encounter from with a globally transmitted parasite:

$$U_{H,g} = (1 - b_H) + b_H \sum_{j}^{n_P} f_{H,j}(1 - I_{g,j}) \qquad \text{(S3.2)}$$

where $f_{H,j}$ is the frequency of parasites of genotype j among the parasites produced by all infected parents (i.e., the global parasite pool, which is the weighted average of all $f_{V,i,j}$). Let $P_{I,g,i,j}$ be the probability that an offspring of genotype g from a mother in infection state i is in infection state j after both infection phases have occurred. It can be shown that

$$P_{I,g,i,j} = \begin{cases} b_V f_{V,i,j} I_{g,j} \dfrac{1 - U_{V,g,i}^{\tau}}{1 - U_{V,g,i}} + b_H f_{H,j} I_{g,j} U_{V,g,i}^{\tau} \dfrac{1 - U_{H,g}^{\tau}}{1 - U_{H,g}} & \text{for } i < n_P + 1 \text{ and } j < n_P + 1 \\ b_H f_{H,j} I_{g,j} \dfrac{1 - U_{H,g}^{\tau}}{1 - U_{H,g}} & \text{for } i = n_P + 1 \text{ and } j < n_P + 1 \\ U_{V,g,i}^{\tau} U_{H,g}^{\tau} & \text{for } i < n_P + 1 \text{ and } j = n_P + 1 \\ U_{H,g}^{\tau} & \text{for } i = n_P + 1 \text{ and } j = n_P + 1 \end{cases}$$

(S3.3)

Let $F_{I,g,j}$ be the frequency of offspring in the population that are of genotype g in infection state j after both infection phases have occurred:

$$F_{I,g,j} = \sum_{i}^{n_P + 1} F_{M,g,i} P_{I,g,i,j} \qquad \text{(S3.4)}$$

Note that no element of selection is included in the calculation of $F_{I,g,j}$.

Let $w_{H,g,j}$ be the fitness of a host of genotype g in infection state j. Let us first consider the fitnesses of uninfected individuals. For MA and IMA models, uninfected individuals have a fitness of 1, i.e., $w_{H,g,n_P+1} = 1$ for all g. For the GFG model, $w_{H,g,n_P+1} = (1-c)^y$ where y is the number of loci at which host g carries at least one resistance allele (resistance alleles are assumed to be dominant to susceptible alleles); c gives the cost of expressing the resistance allele. For all models, the fitnesses of infected individuals can be calculated as, $w_{H,g,j} = (1-v)w_{H,g,n_P+1}$ (for $j < n_P + 1$) where v is the virulence of infection. Mean fitness is given by

$$\overline{w}_H = \sum_{g}^{n_H} \sum_{j}^{n_P+1} F_{I,g,j} w_{H,g,j} \tag{S3.5}$$

The contribution of hosts of genotype g in infection state j to the next generation is

$$C_{g,j} = F_{I,g,j} w_{H,g,j} / \overline{w}_H \tag{S3.6}$$

This contribution may come in the form of sexually or asexually produced offspring. Let σ_g be the fraction of offspring of an individual of genotype g that is produced sexually; the remaining fraction, $1 - \sigma_g$, is produced asexually. The value of σ_g is determined by the alleles carried at the **M** locus; $\sigma_g = \sigma + J_g \delta\sigma/2$ where J_g is the number m alleles carried by genotype g. Considering the entire population, the fraction of offspring in the following generation produced sexually is

$$\sigma_T = \sum_{g}^{n_H} \sum_{j}^{n_P+1} C_{g,j} \sigma_g \tag{S3.7}$$

The frequency of haplotype x amongst the gametes produced by genotype g is given by $\Psi_{g,x}$. This frequency is calculated following the standard rules of Mendelian genetics. The total frequency of this haplotype in the sperm pool is

$$G_x = \sum_{g}^{n_H} \sum_{j}^{n_P+1} C_{g,j} \frac{\sigma_g}{\sigma_T} \Psi_{g,x} \tag{S3.8}$$

Let $K_{h,x,y}$ be an indicator variable that takes a value of one if the combination of haplotype x and haplotype y create a diploid of genotype h but is otherwise zero. The frequency of offspring in the population the following generation that are of genotype h from mothers in infection state i is given by

$$F'_{M,h,i} = C_{h,i}(1-\sigma_h) + \sum_{g}^{n_H} \sum_{x}^{n_G} \sum_{y}^{n_G} C_{g,i} \sigma_g \Psi_{g,x} G_y K_{h,x,y} \tag{S3.9}$$

where n_G is the number of different haplotypes.

Parasites only survive if they successfully infect a host. The frequency of parasite genotype i after selection is given by

$$Q_i = \sum_{g}^{n_H} F_{I,g,j} W_{P,i} \Bigg/ \sum_{g}^{n_H} \sum_{j}^{n_P} F_{I,g,j} W_{P,j} \tag{S3.10}$$

where $W_{P,i}$ is the fitness of parasite i given that it has successfully infected a host. For the MA and IMA models, $W_{P,i}$ =1 for all i. For the GFG model, $W_{P,i} = (1 - k)^y$ where y is the number of loci at which parasite i carries a infectious (virulent) allele; k gives the cost of expressing a infectious (virulent) allele.

All parasites produce a fraction ω of their offspring sexually and the remainder, $1 - \omega$, asexually. As parasites are assumed to live mostly as haploids, syngamy is followed immediately by meiosis. Let $R_{j,x,y}$ be the probability that syngamy and meiosis between haplotypes x and y produces a parasite of genotype j. This probability is calculated following the standard rules of Mendelian genetics. Before mutation, the frequency of genotype j amongst the offspring produced by a parasite of genotype i is

$$q^*_{i,j} = L_{i,j}(1 - \omega) + \omega \sum_{y}^{n_P} Q_y R_{j,i,y} \tag{S3.11}$$

where $L_{i,j} = 1$ for $i = j$ but is zero otherwise. Parasite alleles mutate into the alternate form at rate $\mu = 10^{-5}$. After mutation, the frequency the frequency of frequency of genotype j amongst the offspring produced by a parasite of genotype i is

$$q_{i,j} = \sum_{k}^{n_P} q^*_{i,k} \mu^{d_{j,k}} (1 - \mu)^{s_{j,k}} \tag{S3.12}$$

where $d_{j,k}$ is the number of loci at which genotypes j and k differ and $s_{j,k}$ is the number of loci at which these genotypes are the same. Note that $q_{i,j}$ gives the frequency of genotype j amongst the parasites produced by a mother infected by parasite genotype i, i.e., $f_{V,i,j}[t + 1] = q_{i,j}$. The frequency of parasite j in the global parasite pool the following generation is $f_{H,j}[t+1] = \sum_{i}^{n_P} Q_i q_{ij}$.

Each simulation run was initiated by choosing parasite allele frequencies at random between 0 and 1. Initial host allele frequencies for immunity loci were chosen at random between 0.4 and 0.6. The modifier allele, *m*, always began at a frequency of 0.5. Initial host and parasite genotype distributions were both created assuming no linkage disequilibrium. In the first generation, all hosts were assumed to come from uninfected mothers. During the global transmission phase, this first generation of hosts was exposed to parasites from a global parasite pool defined by the initial parasite genotype distribution. For the MA and IMA models, simulations were run for 4000 generations during which the modifier allele had no phenotypic effect (i.e., "burn-in" period in which $\delta\sigma = 0$). For the GFG model, this period was 15000 generations. Following this burn-in period, the effect of the modifier allele was "turned on" ($\delta\sigma = 0.01$) and the change in the modifier allele's frequency was monitored over the next 1000 to 5000 generations. A minimum of five to ten replicates was run for each parameter combination. If the modifier did not evolve in the same direction in all of these initial replicates, then additional replicates were performed.

The effect of the modifier allele was $\delta\sigma = 0.01$; the amount of investment in sexual reproduction for each genotype was $\sigma_{MM} = \sigma$, $\sigma_{Mm} = \sigma + ½\ \delta\sigma$, and $\sigma_{mm} = \sigma + \delta\sigma$. In the simulations reported, the recombination rate in hosts between loci **M** and **A** was

r_{MA} = 0.1 and between **A** and **B** was r_{AB} = 0.1; in parasites the recombination rate between **A** and **B** was 0.2. Parasites were assumed to be mostly asexual, $\omega = 0.1$. The number of episodes in each infection phase was $\tau = 200$; the desired value for the expected number of global exposures was achieved by adjusting b_V and b_H accordingly. For the GFG model, $c = 0.05v$ and $k = 0.3$.

The patterns displayed in Figure 1 indicate that simulation results were, in a broad sense, consistent with the analytical predictions. The analytical approximations make a number of assumptions including ϕ and $\lambda << 1$ and that there is only a single locus determining infection (see Supporting Information 2). As such, these approximations provide only a heuristic guide and are not expected to explain all of the patterns revealed in simulations, especially in areas of parameter space that deviate substantially from the analytical assumptions.

The most obvious differences between simulations and analytical predictions occurred in the GFG model. The GFG model is intrinsically quite different from the MA and IMA models. In the MA and IMA models, the host population consists of a number of different specialists. Each host genotype is specialized at resisting a different subset of parasite genotypes. In the GFG model, the host population consists of a mixture of specialists and generalists. Resistant host genotypes can be considered generalists in that they are able to resist a broader array of parasite genotypes than are susceptible host genotypes. Polymorphism is maintained by a cost of the generalist strategy, i.e., a cost of resistance in hosts and costs of infectiousness in parasites.

Although the number of exposures is not expected to have a biased effect on the fitnesses of different specialist genotypes in MA or IMA models, it does have a biased

effect on the fitnesses of generalists versus specialist strategies in the GFG model. When exposure rate is very high, even a host carrying a resistance allele is likely to encounter at least one parasite capable of infecting it, in which case the benefit of the resistance allele relative to its intrinsic cost is low. When exposure rate becomes very high (e.g., $\lambda = 10$) there is a qualitative shift in the system in the GFG model that is not captured by the analytical approximations that assume $\lambda << 1$. Specifically, the system shifts from one in which infectious parasite genotypes occur at high frequency and resistant hosts are quite common to one in which infectious parasite genotypes are rare and resistant hosts are less frequent. Examination of the single-species selection coefficients generated by host-parasite simulations indicates that both genotypic and similarity selection are stronger when exposure rates are very high. Most importantly, the ratio of the square of the non-linear component of genotypic selection to the strength of similarity selection, $\iota_{\mathbf{A}}^2/\theta_{\mathbf{A}}$, increases when λ is large, which tends to select against sex.

Typically, increasing ϕ increases $\theta_{\mathbf{A}}$ so that sex is more likely to be favoured. However, in some cases increasing ϕ selects against sex in the GFG model. Considering both global and maternal transmission, the total number of exposures is $\lambda(1 + \phi)$. Consequently, changes in ϕ can have a substantial impact on the total number of exposures when ϕ is large. Such a change in the total number of exposures can affect overall host-parasite dynamics including the distributions of host and parasite genotypes, which are key determinants of the realized selection coefficients. Thus, the effect of ϕ on the selection coefficients is not always straightforward. Occasionally, an increase in ϕ reduces the average value of $\theta_{\mathbf{A}}$ so that similarity selection declines in strength relative to genotypic selection, which acts against sex. In other cases, increasing ϕ increases $\theta_{\mathbf{A}}$ as

expected, thus favouring sex, but also causes an increase in the magnitude of the non-linear component of genotypic selection, $|\iota_{\mathbf{A}}|$, which results in selection against sex. Increases in the strength of genotypic selection are expected to be especially important to the evolution of sex when the baseline level of sex is low. Selection against sex with high values of ϕ tends to occur at very low baseline levels of sex, $\sigma = 0$.

Supporting Information 3 - Tables

	Parasite Genotype			
Host Genotype	*AB*	*Ab*	*aB*	*ab*
AABB	1	0	0	0
AABb	*H*	*H*	0	0
AAbb	0	1	0	0
AaBB	*H*	0	*H*	0
AaBb	H^2	H^2	H^2	H^2
Aabb	0	*H*	0	*H*
aaBB	0	0	1	0
aaBb	0	0	*H*	*H*
aabb	0	0	0	1

Table S3.1. Probability of infection of host upon exposure to parasite. Two-locus Matching Alleles (MA) model. H is the probability that a heterozygous locus acts like a match. The simulations performed used $H = 0.75$. Note that H is related to the parameter d_R in Table 2 by the equation $H = ½ + d_R$, i.e., in the simulations $d_R = 0.25$.

	Parasite Genotype			
Host Genotype	*AB*	*Ab*	*aB*	*ab*
AABB	0	1	1	1
AABb	$1 - H$	$1 - H$	1	1
AAbb	1	0	1	1
AaBB	$1 - H$	1	$1 - H$	1
AaBb	$(1 - H)^2$	$(1 - H)^2$	$(1 - H)^2$	$(1 - H)^2$
Aabb	1	$1 - H$	1	$1 - H$
aaBB	1	1	0	1
aaBb	1	1	$1 - H$	$1 - H$
aabb	1	1	1	0

Table S3.2. Probability of infection of host upon exposure to parasite. Two-locus Inverse Matching Alleles (IMA) model. H is the probability that a heterozygous locus acts like a match. The simulations reported used $H = 0.75$. Note that H is related to the parameter d_R in Table 2 by the equation $1 - H = ½ + d_R$, i.e., in the simulations $d_R = -0.25$.

	Parasite Genotype			
Host Genotype	*AB*	*Ab*	*aB*	*ab*
AABB	0	0	0	1
AABb	0	0	0	1
AAbb	0	0	1	1
AaBB	0	0	0	1
AaBb	0	0	0	1
Aabb	0	0	1	1
aaBB	0	1	0	1
aaBb	0	1	0	1
aabb	1	1	1	1

Table S3.3. Probability of infection of host upon exposure to parasite. Two-locus Gene For Gene (GFG) model.
